# Regulation of vanillate and syringate catabolism by a MarR-type transcriptional regulator DesR in *Sphingobium* sp. SYK-6

**DOI:** 10.1038/s41598-019-54490-7

**Published:** 2019-12-02

**Authors:** Takuma Araki, Shusuke Umeda, Naofumi Kamimura, Daisuke Kasai, Shuta Kumano, Tomokuni Abe, Chika Kawazu, Yuichiro Otsuka, Masaya Nakamura, Yoshihiro Katayama, Masao Fukuda, Eiji Masai

**Affiliations:** 1 Department of Bioengineering, Nagaoka University of Technology, Nagaoka, Niigata 940-2188 Japan; 2 Forestry and Forest Products Research Institute, Tsukuba, Ibaraki 305-8687 Japan; 3 College of Bioresource Sciences, Nihon University, Fujisawa, Kanagawa 252-0880 Japan; 4 Present Address: Department of Biological Chemistry, Chubu University, Kasugai, Aichi 487-8501 Japan

**Keywords:** Metabolism, Gene expression, Bacterial transcription

## Abstract

Vanillate and syringate are major intermediate metabolites generated during the microbial degradation of lignin. In *Sphingobium* sp. SYK-6, vanillate is O demethylated to protocatechuate by LigM; protocatechuate is then catabolized via the protocatechuate 4,5-cleavage pathway. Syringate is O demethylated to gallate by consecutive reactions catalyzed by DesA and LigM, and then gallate is subjected to ring cleavage by DesB. Here, we investigated the transcriptional regulation of *desA*, *ligM*, and *desB* involved in vanillate and syringate catabolism. Quantitative reverse transcription-PCR analyses indicated that the transcription of these genes was induced 5.8–37-fold in the presence of vanillate and syringate. A MarR-type transcriptional regulator, SLG_12870 (*desR*), was identified as the gene whose product bound to the *desB* promoter region. Analysis of a *desR* mutant indicated that the transcription of *desB*, *ligM*, and *desR* is negatively regulated by DesR. Purified DesR bound to the upstream regions of *desB*, *ligM*, and *desR*, and the inverted repeat sequences similar to each other in these regions were suggested to be essential for DNA binding of DesR. Vanillate and syringate inhibited DNA binding of DesR, indicating that these compounds are effector molecules of DesR. The transcription of *desA* was found to be regulated by an as-yet unidentified regulator.

## Introduction

Lignin, a major component of plant cell walls, is the most abundant aromatic polymer in nature, and its biodegradation makes a major contribution to the Earth’s carbon cycle^1^. The effective use of lignin as biomass is desirable because it is an enormously abundant and renewable aromatic resource^2^. Lignin is formed by the oxidative coupling of three types of *p*-hydroxyphenylpropanoids, called monolignols: coniferyl alcohol, sinapyl alcohol, and *p*-coumaryl alcohol^3^. Lignin structure differs depending on the type of plant that produced it. Gymnosperm (softwood) lignins contain guaiacyl (G)-units derived from coniferyl alcohol with small amounts of *p*-hydroxyphenyl (H)-units derived from *p*-coumaryl alcohol; angiosperm (hardwood) lignins contain syringyl (S)-units derived from sinapyl alcohol and G-units; while monocot (grass) lignins contain G-units, S-units, and more H-units than gymnosperm lignins^4,5^.

Lignin depolymerization is most extensively characterized in white-rot fungi and there is some characterization in bacteria. Lignin peroxidase, manganese peroxidase, versatile peroxidase, and laccase, secreted by white-rot fungi are well characterized enzymes involved in lignin depolymerization^6–8^. Dye-decolorizing peroxidases secreted by some bacteria are thought to be partially involved in lignin depolymerization^9,10^. The resulting low molecular-weight aromatic compounds are assumed to be mineralized by bacteria, and various enzymes involved in this process have been reported^9,11,12^. In particular, bacterial catabolic systems have attracted great attention due to their ability to convert aromatic compounds obtained from the chemical decomposition of lignin into industrially useful platform chemicals^13–15^.

Bacterial catabolism of lignin-derived aromatic compounds has been intensively studied in the alphaproteobacterium *Sphingobium* sp. SYK-6^12,16^. SYK-6 is able to grow on lignin-derived biaryls which have β-O-4, β-5, 5–5, and β-1 linkages as its sole carbon and energy source, as well as monoaryls such as ferulate, using a number of specific enzymes. These aromatic compounds derived from G- and S-lignins are degraded through vanillate (VA) and syringate (SA), respectively^12,16^. In addition to SYK-6 catabolism, it has been reported that lignin-derived aromatic compounds, including ferulate, sinapate, vanillin, and syringaldehyde, are degraded via VA and SA in many microorganisms^9,17–20^. Therefore, VA and SA are considered to be the key intermediate metabolites in the microbial degradation of lignin.

**Figure 1:**
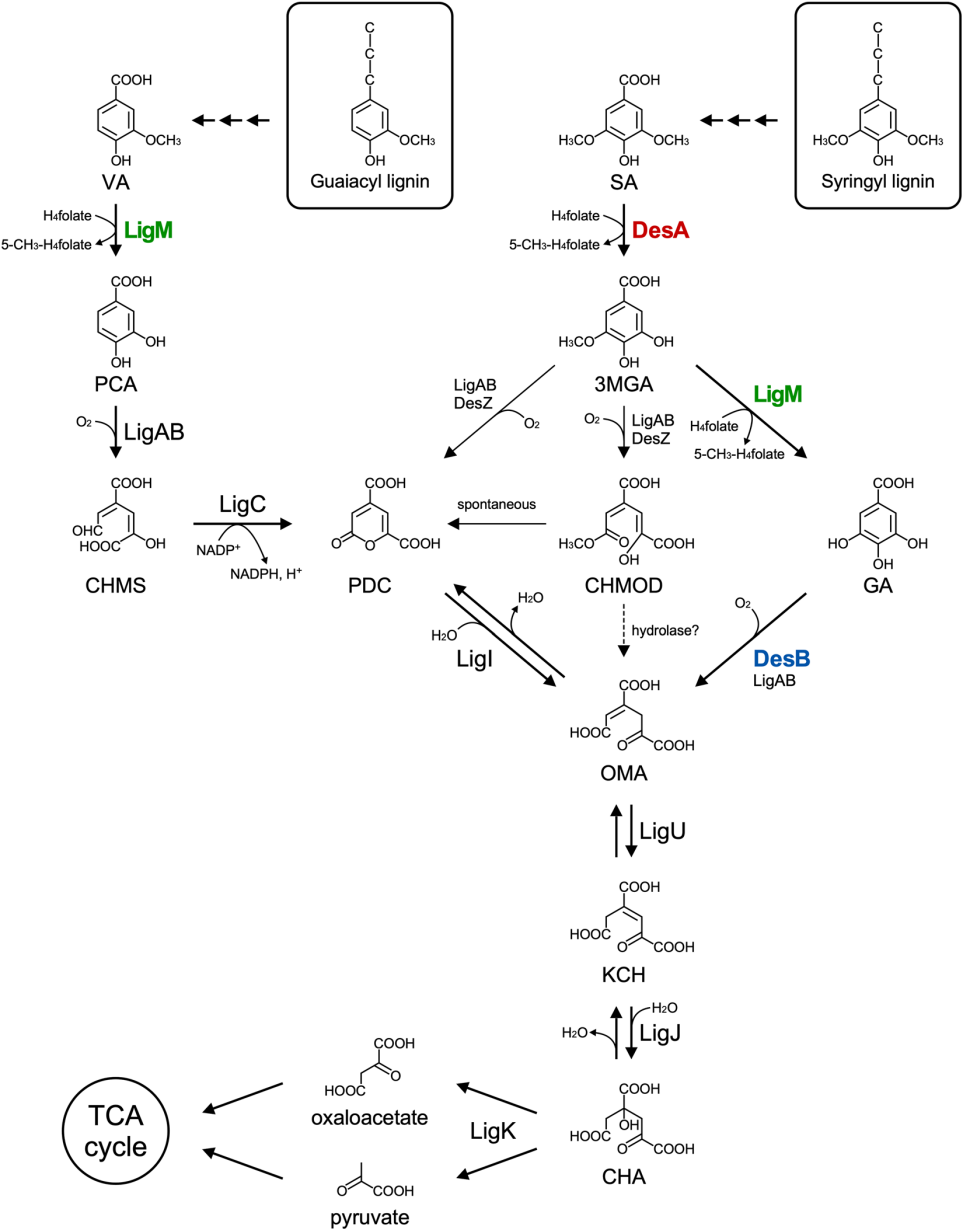
Catabolic pathways for vanillate and syringate in *Sphingobium* sp. SYK-6. The catabolic pathways proposed in our previous study^12,27^ have been updated based on a recent report^49^. Enzymes: LigM, vanillate/3MGA *O*-demethylase; LigA and LigB, small and large subunits, respectively, of PCA 4,5-dioxygenase; LigC, CHMS dehydrogenase; LigI, PDC hydrolase; LigU, OMA delta-isomerase; LigJ, KCH hydratase; LigK, CHA aldolase; DesA, syringate *O*-demethylase; DesZ, 3MGA 3,4-dioxygenase; DesB, gallate dioxygenase. Abbreviations: VA, vanillate; PCA, protocatechuate; CHMS, 4-carboxy-2-hydroxymuconate-6-semialdehyde; PDC, 2-pyrone-4,6-dicarboxylate; OMA, 4-oxalomesaconate; KCH, 2-keto-4-carboxy-3-hexenedioate; CHA, 4-carboxy-4-hydroxy-2-oxoadipate; SA, syringate; 3MGA, 3-*O*-methylgallate; GA, gallate; CHMOD, 4-carboxy-2-hydroxy-6-methoxy-6-oxohexa-2,4-dienoate.

Degradation of VA by bacteria is initiated either by the oxygenase-type or by the tetrahydrofolate (H_4_folate)-dependent O demethylation system. An oxygenase-type system, VA *O*-demethylase (VanAB), which is composed of an oxygenase (VanA) and a reductase (VanB) and is known as a class IA oxygenase, is widely distributed in Gram-negative bacteria, such as *Pseudomonas* strains^21,22^ and *Acinetobacter baylyi* ADP1^23^, as well as Gram-positive bacteria including *Corynebacterium glutamicum* ATCC 13032^24^ and *Rhodococcus jostii* RHA1^25^. In these bacteria, VA is converted to protocatechuate (PCA) by VanAB, and then PCA is metabolized via the PCA 3,4-cleavage pathway. In contrast, VA is converted to PCA by H_4_folate-dependent VA/3-*O*-methylgallate (3MGA) *O*-demethylase (LigM) in SYK-6 (Fig. 1)^26^. In this reaction, LigM catalyzes the direct transfer of the methyl moiety of the methoxy group of VA to H_4_folate, generating 5-CH_3_-H_4_folate that flows into the one-carbon (C_1_) metabolism^12,16^. The resulting PCA is further metabolized via the PCA 4,5-cleavage pathway involving PCA 4,5-dioxygenase (LigAB) (Fig. 1)^27^. On the other hand, SA is converted to 3MGA by another H_4_folate-dependent *O*-demethylase (DesA), which shows 49% amino acid sequence identity with LigM in SYK-6 (Fig. 1)^28^. The resulting 3MGA is metabolized via three different pathways: (i) O demethylation of 3MGA by LigM followed by the aromatic ring cleavage of the resulting gallate (GA) by GA dioxygenase (DesB) and LigAB, generating an intermediate metabolite of the PCA 4,5-cleavage pathway, 4-oxalomesaconate (OMA); (ii and iii) the ring cleavage of 3MGA by 3MGA 3,4-dioxygenase (DesZ) and LigAB, generating 4-carboxy-2-hydroxy-6-methoxy-6-oxohexa-2,4-dienoate [CHMOD (ii)], which is converted to OMA by a hydrolase, and 2-pyrone-4,6-dicarboxylate (iii), an intermediate metabolite of the PCA 4,5-cleavage pathway (Fig. 1)^29–31^. A mutant analysis indicated that the GA cleavage pathway involving LigM and DesB (pathway i) plays a crucial role in SA catabolism^31^. The SA catabolic pathway has so far been genetically and biochemically characterized only in SYK-6. Recently, the SA catabolic pathway and its pathway genes were reported for *Novosphingobium aromaticivorans* DSM 12444^32^. In this strain, SA is first O demethylated by DesA, then the resultant 3MGA is ring-cleaved by LigAB to produce CHMOD, which is converted to OMA by consecutive reactions of demethylation and *cis*-*trans* isomerization catalyzed by DesC and DesD, respectively^32^. Therefore, in DSM 12444, SA is catabolized without passing through GA produced by the O demethylation of 3MGA.

Although the SYK-6 genes involved in the catabolism of lignin-derived aromatic compounds have been extensively investigated, their transcriptional regulatory systems are largely unknown except for the regulation of the PCA 4,5-cleavage pathway genes and the ferulate catabolic genes^33,34^. In other bacteria, the transcriptional regulation of *vanAB* has been documented. The transcription of *vanAB* in *A*. *baylyi* ADP1/*Caulobacter crescentus* and *C. glutamicum* is negatively regulated by VanR belonging to GntR-type and PadR-like transcriptional regulators, respectively^35–37^. VA acts as an effector molecule for the systems in *C. crescentus* and *C. glutamicum*^36,37^. In the case of SYK-6, *ligM* (SLG_12740), *desA* (SLG_25000), and *desB* (SLG_03330), involved in VA/SA, SA, and SA catabolism, respectively, are scattered throughout the chromosome^12,16^. However, it is thought that these genes are synchronously expressed which enables them to efficiently catabolize VA and SA. Because VA and SA are the key intermediate metabolites of lignin biodegradation, elucidation of the transcriptional regulation of the VA and SA catabolic genes is essential if the complete regulatory system of bacterial lignin catabolism is to be understood.

In the present study, we focused on the transcriptional regulation of *ligM*, *desA*, and *desB* in SYK-6 to determine the regulatory system for VA and SA catabolism. The regulatory system was characterized through the identification of *desR*, which encodes a MarR-type transcriptional regulator.

## Results

### Transcriptional analysis of the VA and SA catabolic genes

In order to investigate the inducibility of the conversion of VA and SA in *Sphingobium* sp. SYK-6, the rate of VA and SA conversion by SYK-6 was measured using cells grown in Wx minimal medium^34^ containing 10 mM sucrose, 10 mM glutamate, 0.13 mM methionine, and 10 mM proline (Wx-SEMP; the non-inducing condition) and Wx-SEMP plus 5 mM VA or SA (the inducing condition). The rate of conversion of 200 µM VA or SA by cells grown under the inducing conditions (ca. 8.7–9.3 µM/min and 7.0–7.9 µM/min, respectively) was approximately 6.5- to 6.9- and 3.2- to 3.6-times higher than that of cells grown under the non-inducing condition (ca. 1.4 µM/min and 2.2 µM/min) (Fig. S1). These results indicate that the conversion of VA and SA occurred after incubation in both non-inducing condition and inducing condition, however, the effect was more pronounced when SYK-6 cells were incubated with VA or SA prior to assays.

**Figure 2:**
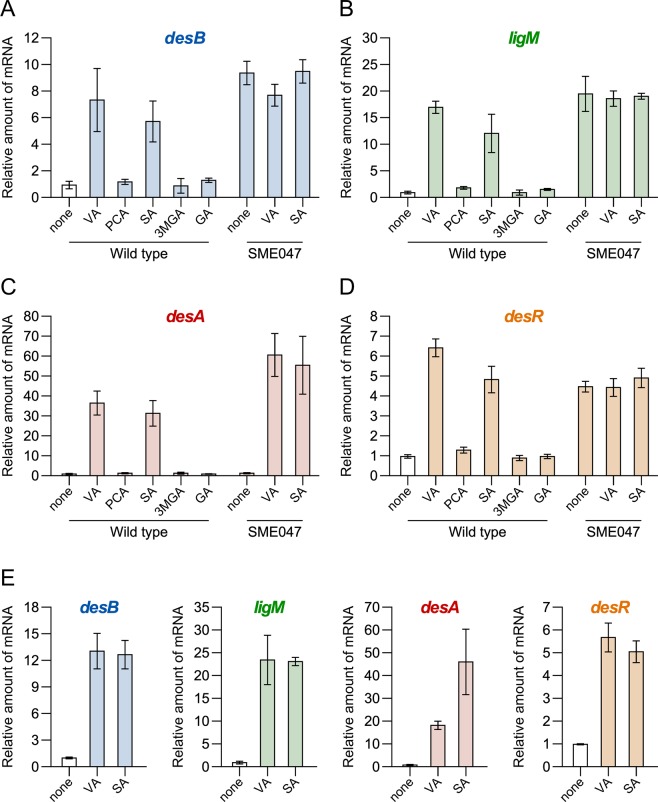
qRT-PCR analysis of the expression of *desB*, *ligM*, *desA*, and *desR*. (**A–D**) qRT-PCR analysis of *desB*, *ligM*, *desA*, and *desR*, respectively, in SYK-6 and a SLG_12870 mutant (SME047). (**E**) qRT-PCR analysis of *desB*, *ligM*, *desA*, and *desR* in a *desA ligM* double mutant (SME021). Total RNAs were isolated from the cells of SYK-6, SME047, and SME021 grown in Wx-SEMP supplemented with or without 5 mM VA, PCA, SA, 3MGA, or GA. Relative amounts of mRNA indicate fold increases over the amount of mRNA in SYK-6 cells of grown in Wx-SEMP (level of 1.0; white bar). Values for each amount of mRNA were normalized to the level of 16S rRNA (Table S5). Each value is the average ± the standard deviation (error bars) of three independent experiments.

To examine whether the inducible conversion of VA and SA in SYK-6 is caused at the transcriptional level, we performed quantitative reverse transcription-PCR (qRT-PCR) analyses of *desB*, *ligM*, and *desA*. The transcribed amounts of *desB*, *ligM*, and *desA* were measured based on total RNA isolated from SYK-6 cells grown in Wx-SEMP, Wx-SEMP plus VA, and Wx-SEMP plus SA. This analysis showed that under the inducing conditions compared with the non-inducing condition, the levels of *desB*, *ligM*, and *desA* transcribed increased 5.8- to 7.4-, 12- to 17-, and 32- to 37-fold, respectively, (Fig. 2A–C). These results indicate that the conversion of VA and SA in SYK-6 cells is regulated at the level of transcription. In contrast, the transcription levels of these genes in SYK-6 cells grown in the presence of intermediate metabolites of VA or SA, such as PCA, 3MGA, and GA, were almost equal to those of cells grown under the non-inducing condition (Fig. 2A–C). Therefore, VA and SA appear to act as inducers of *desB*, *ligM*, and *desA*.

An internal deletion mutant of *desA* and *ligM* (SME021; formerly DDAM)^26^, deficient in the conversion of both VA and SA, was also subjected to qRT-PCR analysis. In SME021 cells, the transcription levels of *desB*, *ligM*, and *desA* increased 13-, 23-, and 18- to 46-fold, respectively, under the inducing conditions compared with the non-inducing condition (Fig. 2E). Therefore, we concluded that VA and SA are the inducers of *desB*, *ligM*, and *desA*.

### Identification of a transcriptional regulator of the VA and SA catabolic genes

**Figure 3:**
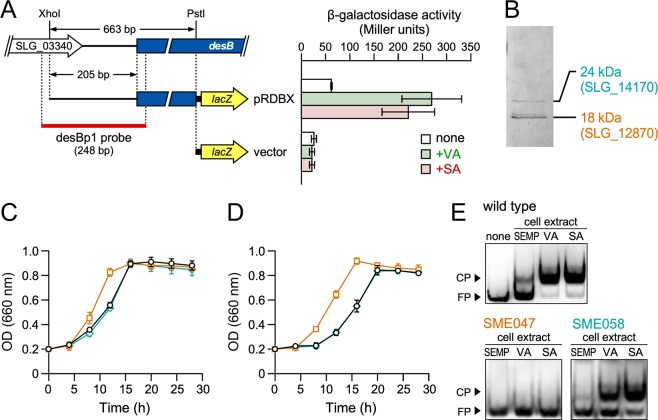
Isolation and characterization of SYK-6 proteins bound to the upstream region of *desB*. (**A**) Promoter analysis of *desB*. The DNA fragments used for the promoter analysis of *desB* and EMSA are shown on the left. β-galactosidase activities of SYK-6 cells harboring pRDBX or pPR9TZ (vector) grown in the presence and absence of 5 mM VA or SA are shown to the right. Each value is the average ± the standard deviation (error bars) of three independent experiments. (**B**) Isolation of SYK-6 proteins bound to the upstream region of *desB*. A cell extract of SYK-6 grown in LB was incubated with a biotin-labeled desBp1 probe. The proteins bound to the fragment were separated by SDS-PAGE. The two protein bands detected were tryptic digested and analyzed by peptide mass fingerprinting. (**C** and **D**) Growth of a SLG_12870 mutant and a SLG_14170 mutant on VA or SA. Cells of SYK-6 (black circles), a SLG_12870 mutant (SME047; orange squares), and a SLG_14170 mutant (SME058; cyan diamonds) were incubated in Wx medium containing 5 mM VA (**C**) or 5 mM SA (**D**), and OD_660_ was periodically monitored. Each value is the average ± the standard deviation (error bars) of three independent experiments. (**E**) EMSAs of SYK-6 cell extracts using a *desB* promoter region probe. A digoxigenin-labeled desBp1 probe (500 pM) was incubated in the presence and absence of the extracts (4  µg of protein) of wild type, SME047, and SME058 cells grown in Wx-SEMP, Wx-SEMP + VA, and Wx-SEMP + SA. CP, DNA-protein complex; FP, free probe. The cropped gel images are shown, and the full-length gels are presented in Fig. S7.

SYK-6 cells harboring pRDBX (Table S3), which carries a 663-bp fragment containing the region 205-bp upstream of *desB* in a promoter probe vector pPR9TZ carrying *lacZ* as the reporter gene (Table S3), were grown under the inducing conditions. These cells showed ca. 3.5- to 4.3-times higher promoter activity than cells grown under the non-inducing condition (Fig. 3A). Therefore, the upstream region of *desB* appeared to contain the promoter region essential for the transcriptional regulation of *desB*. In order to identify this transcriptional regulator of *desB*, we performed DNA-affinity purification using streptavidin-conjugated magnetic beads. After a cell extract of SYK-6 grown in lysogeny broth (LB) was incubated with a biotin-labeled 248-bp fragment containing the putative *desB* promoter region (desBp1 probe, Fig. 3A), proteins bound to this fragment were recovered from the magnetic beads. The resulting proteins were separated by sodium dodecyl sulfate-polyacrylamide gel electrophoresis (SDS-PAGE). Two proteins, of 18 kDa and 24 kDa in size, were recovered (Fig. 3B) and identified as SLG_12870 and SLG_14170, respectively, by peptide mass fingerprinting using matrix-assisted laser desorption/ionization time-of-flight mass spectrometry (MALDI-TOF-MS). BLAST searches showed that SLG_12870 had similarity with MarR-type transcriptional regulators, however their similarities were relatively low (E-value score, >1.01E–79) (Table S1). SLG_14170 showed moderately high similarities (E-value score <2.58E–139) with many other LysR-type transcriptional regulators (Table S2).

A SLG_12870 mutant (SME047) and a SLG_14170 mutant (SME058) were created by homologous recombination to examine the involvement of these genes in the regulation of VA and SA catabolism (Fig. S2). SME047 grew significantly faster than the wild type in Wx medium containing 5 mM VA or SA, whereas the growth of SME058 was almost the same as that of the wild type (Fig. 3C,D). SME047 harboring a plasmid carrying SLG_12870 (pJBdR) showed a delay in growth on VA compared with SME047 harboring a vector (pJB866) (Fig. S3). Furthermore, the introduction of pJBdR in SYK-6 caused the retardation of its growth on VA. These results clearly indicate that the changed phenotype of SME047 was caused by the disruption of SLG_12870. In order to confirm that the gene products of SLG_12870 and SLG_14170 bind to the *desB* promoter region, an electrophoretic mobility shift assay (EMSA) was performed using cell extracts of the wild type, SME047, and SME058 with a digoxigenin-labeled desBp1 probe (Fig. 3A). A shift band was observed when using the extracts of the wild type and SME058 cells grown in Wx-SEMP, Wx-SEMP plus VA, and Wx-SEMP plus SA (Fig. 3E). However, no shift band was detected with any of the SME047 cell extracts. These results suggest that the gene product of SLG_12870 binds to the *desB* promoter region and negatively regulates at least one of the VA and SA catabolic genes. Therefore, we designated SLG_12870 as *desR*. The gene product of SLG_14170 seems to have been obtained by chance during the process of DNA-affinity purification.

### Transcriptional analysis of VA and SA catabolic genes and *desR* in a *desR* mutant

qRT-PCR analyses of *desB*, *ligM*, and *desA* in SME047 (an internal deletion mutant of *desR*) cells were performed. The transcription levels of *desB* and *ligM* in SME047 cells grown under the non-inducing condition increased 9.4- and 20-fold, respectively, compared with those of the wild-type cells grown under the same condition (Fig. 2A,B). In addition, the transcription levels of *desB* and *ligM* were almost the same between SME047 cells grown under the inducing conditions and the non-inducing condition. It is therefore apparent that the transcription of *desB* and *ligM* is dominantly and negatively regulated by DesR. On the other hand, the transcription levels of *desA* in SME047 and SYK-6 cells grown under the non-inducing condition were almost identical, and *desA* transcription was similarly induced in SME047 and wild-type cells (Fig. 2C). These results indicate that an unidentified transcriptional regulator, different from DesR, is involved in the transcriptional control of *desA*.

In general, it is known that MarR-type transcriptional regulators regulate their own gene expression by repression/derepression^38^. The transcription level of *desR* in SYK-6 cells increased 4.8- to 6.4-fold under the inducing conditions compared with the non-inducing condition (Fig. 2D). In contrast, the transcription level of *desR* in SME047 cells grown under the non-inducing condition was equivalent to that of the wild-type cells grown under the inducing conditions (Fig. 2D). Therefore, DesR also regulates its own gene expression by repression/derepression. Based on the similar transcription profiles of *desR* seen in the wild-type and SME021 (*desA ligM* mutant) cells, VA and SA were determined to be the inducers of *desR* (Fig. 2E).

### Determination of the transcription start sites of *desB* and *ligM*

Primer extension analysis demonstrated that the transcription start sites of *desB* and *ligM* were mapped to the A residue located 109 nucleotides upstream of the initiation codon of *desB* and the C residue located 68 nucleotides upstream of the initiation codon of *ligM*, respectively (Fig. S4). The putative −35 and −10 sequences, which are somewhat similar to the conserved sequence of the σ^70^-dependent promoter of *E*. *coli*, were found upstream of each transcription start site of *desB* and *ligM*. In addition, there were 15-bp inverted repeat (IR) sequences, IR-B (GTTTGTGTCACATAC) and IR-M (GTTTGTGTAACATAC), at positions +23 to +37 and −40 to −26 relative to the transcription start sites of *desB* and *ligM*, respectively (Fig. S4). IR-B and IR-M exhibit high sequence similarity to each other (14 out of 15 matches).

### Binding of DesR to the upstream regions of *desB*, *ligM*, and *desR*

*desR* fused with a His tag at the 5′ terminus was expressed under the control of the *cspA* promoter in *E*. *coli* BL21(DE3) harboring pCIdR (Table S3). SDS-PAGE analysis identified the production of a 20-kDa protein (Fig. S5A). The molecular mass of this protein was close to the theoretical molecular weight (21,522) of His-tag fused DesR. DesR was purified to near homogeneity using Ni affinity chromatography (Fig. S5A). The native molecular mass of DesR was estimated to be 42 kDa by size exclusion chromatography (Fig. S5B,C). This result suggests that DesR in solution forms a homodimer, which is a common feature of MarR-type transcriptional regulators^38,39^.

**Figure 4:**
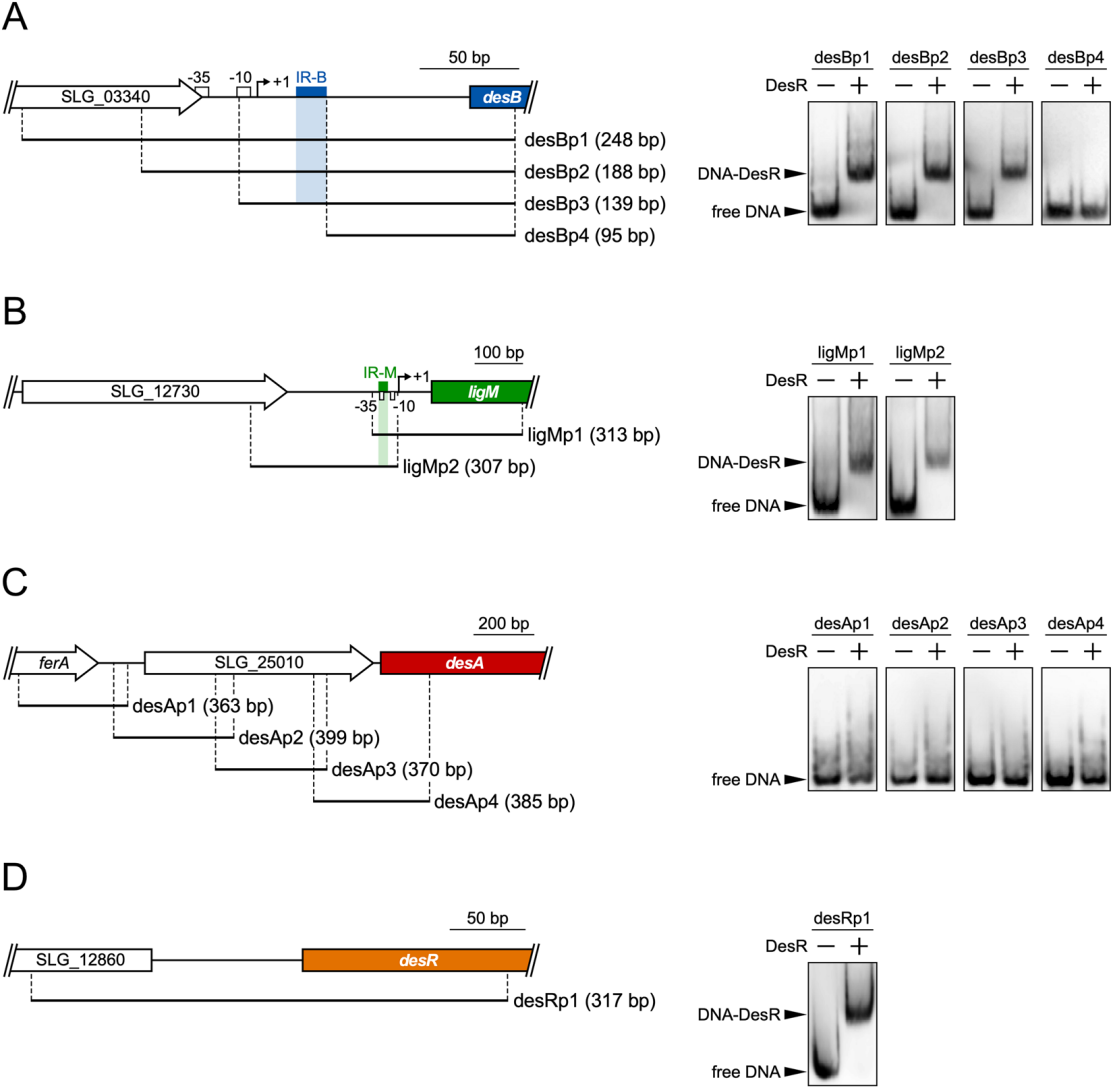
Binding of DesR to the upstream regions of *desB*, *ligM*, *desA*, and *desR*. (**A–D**) The panels on the left show the DNA fragments carrying the upstream regions of *desB*, *ligM*, *desA*, and *desR*, respectively, used for the EMSAs. The panels on the right show the results of the EMSAs of the binding of DesR to each DNA probe. Digoxigenin-labeled DNA probes (500 pM) were incubated in the presence (+) and absence (−) of purified DesR (240 nM). The cropped gel images are shown, and the full-length gels are presented in Fig. S7.

EMSA was performed to examine whether DesR binds to the upstream regions of *desB*, *ligM*, and *desA*. Digoxigenin-labeled DNA fragments containing various upstream regions of *desB* (desBp1–desBp4), *ligM* (ligMp1 and ligMp2), and *desA* (desAp1−desAp4) were incubated with purified DesR (Fig. 4A–C). Shift bands showing the formation of a DNA−DesR complex were observed for the desBp1, desBp2, and desBp3 probes while the desBp4 probe resulted in no shift band (Fig. 4A). These results suggest that IR-B is essential for the binding of DesR to the *desB* promoter region. Similarly, the formation of a DNA−DesR complex was observed for the ligMp1 and ligMp2 probes containing IR-M (Fig. 4B). In contrast, no shift bands were obtained with any of the probes containing the *desA* upstream regions (desAp1–desAp4) (Fig. 4C). In addition, no DNA sequence motifs similar to IR-B or IR-M were found in the desAp1–desAp4 regions. Based on the results obtained by EMSA, the absence of IR-B/M-like motifs upstream of *desA*, and the fact that the disruption of *desR* did not affect *desA* transcription (Fig. 2C), it can be concluded that DesR is not involved in the transcriptional regulation of *desA*. To investigate whether DesR binds to the *desR* upstream region, EMSA was performed using a 317-bp probe containing an SLG_12860–*desR* intergenic region (Fig. 4D). A shift band was observed, demonstrating that DesR binds to the *desR* upstream region. We found an IR sequence, IR-R (GTATGCTACGCTTAC), which showed similarity with IR-B and IR-M and was located 76 to 62 nucleotides upstream of the *desR* start codon (Fig. S6).

**Figure 5:**
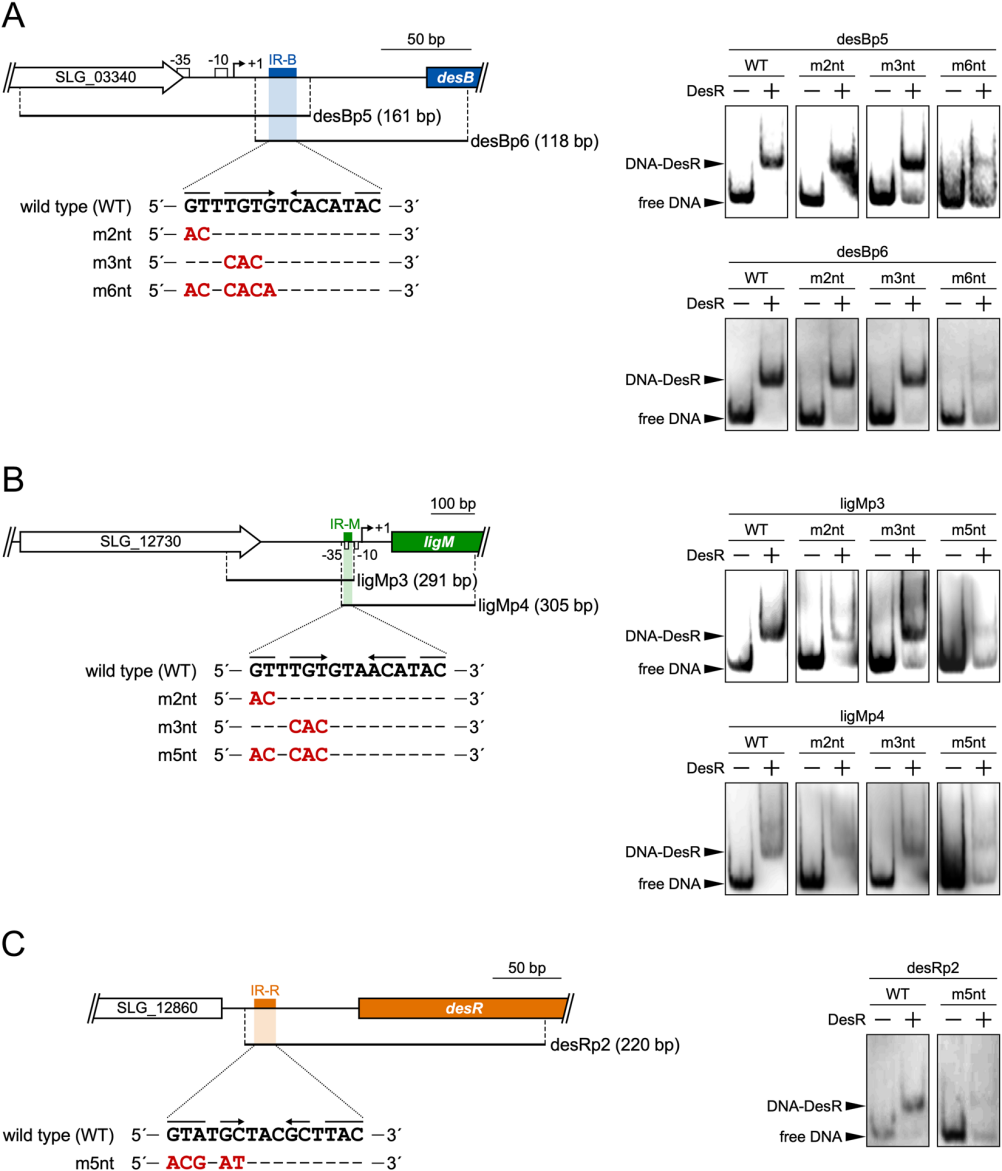
Binding of DesR to the *desB*, *ligM*, and *desR* upstream regions containing mutated IR sequences. (**A–C**) The panels on the left show the DNA fragments carrying the upstream regions of *desB*, *ligM*, and *desR* that contained mutated IR-B, IR-M, and IR-R, respectively, used for the EMSAs. The panels on the right show the results of the EMSAs of the binding of DesR to each DNA probe. Digoxigenin-labeled mutated DNA probes (500 pM) were incubated in the presence (+) and absence (−) of purified DesR (240 nM). In the presence of DesR, the intensities of the shift bands of specific probes were weak for unknown reasons. The cropped gel images are shown, and the full-length gels are presented in Fig. S7.

To clarify whether IR-B, IR-M, or IR-R are essential for DesR binding, we examined the binding of DesR to DNA probes containing mutated IR-B, IR-M, and IR-R, respectively (Fig. 5). An EMSA using desBp5 and desBp6 probes containing IR-B at the 3′ and 5′ ends of the probes, respectively, showed the formation of a DNA-DesR complex (Fig. 5A). In contrast, when using desBp5 and desBp6 probes containing 2-base (m2nt), 3-base (m3nt), and 6-base (m6nt) mutations in the 5′ half of IR-B, the formation of shift bands was increasingly inhibited as the number of mutations increased (Fig. 5A). Similar results were obtained by an EMSA using ligMp3 and ligMp4 probes containing mutated IR-M (Fig. 5B) and a desRp2 probe containing mutated IR-R (Fig. 5C). Shift bands were still observed even when using the m6nt and m5nt probes. Since similar sequences of IR-B and IR-M were not found in these probes, it suggests that the 3′ half site to some extent retained the ability to bind to DesR. Taken together these results strongly suggest that IR-B, IR-M, and IR-R are essential for the DNA binding of DesR.

### Identification of the effector molecules of DesR

**Figure 6:**
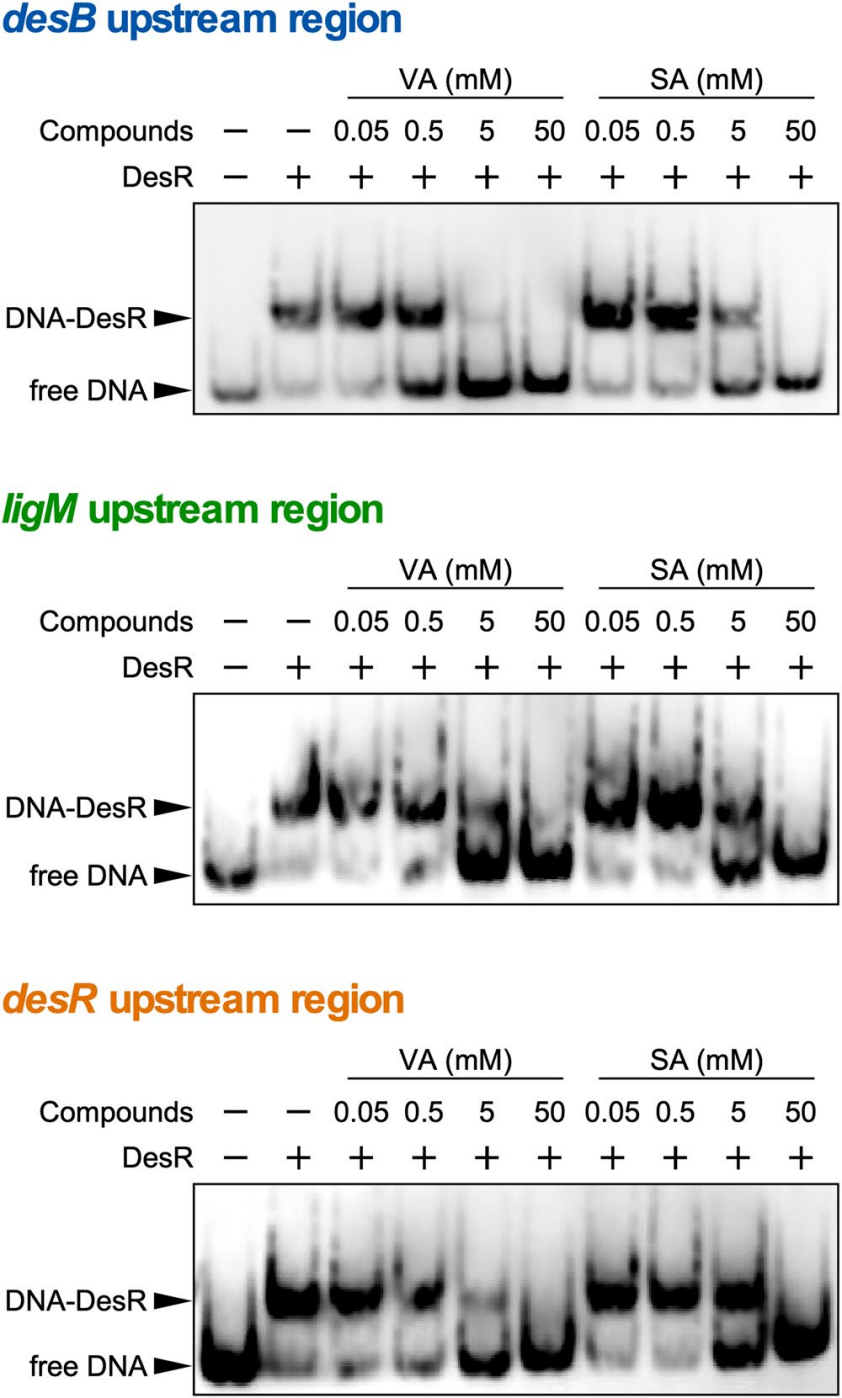
Identification of the effector molecules of DesR. Purified DesR (24 nM) and DNA probes (500 pM) containing the upstream regions of *desB* (desBp2), *ligM* (ligMp1), and *desR* (desRp1) were incubated in the presence (0.05, 0.5, 5, or 50 mM) or absence of VA or SA. The cropped gel images are shown, and the full-length gels are presented in Fig. S7.

qRT-PCR analyses using the *desA ligM* double mutant (SME021) indicated that VA and SA are inducers of *desB*, *ligM*, and *desR* (Fig. 2E). In order to investigate the effect of VA and SA on the DNA binding of DesR, EMSAs were carried out using DesR and probes containing the upstream regions of *desB*, *ligM*, and *desR* in the presence of VA or SA (0.05, 0.5, 5, and 50 mM). In the presence of VA, shift bands of these three probes decreased in a concentration-dependent manner, and disappeared completely in the presence of 50 mM VA (Fig. 6). A decrease in the shift bands of the three probes was also observed in a concentration-dependent manner in the presence of SA. However, the effect of SA on the DNA binding of DesR was weaker than that of VA (Fig. 6). These results indicate that VA and SA are effector molecules of DesR although they have different affinities.

## Discussion

From the results of this study it can be concluded that the transcription of *ligM*, *desB*, and *desR* is negatively regulated by a MarR-type transcriptional regulator, DesR. Based on the general properties of MarR-type transcriptional regulators, the binding of VA or SA to DesR (DesR was suggested to have a higher affinity for VA than SA.) is likely to cause derepression of *ligM*, *desB*, and *desR*. The DesR binding sites, IR-M and IR-B, were found to be located at positions −40 to −26 and +23 to +37 from the transcriptional start sites of *ligM* and *desB*, respectively (Fig. S4). Because IR-M overlaps with the −35 region, DesR appears to interfere with the binding of RNA polymerase to the *ligM* promoter region. Furthermore, DesR is likely to hamper the progression of RNA polymerase in the *desB* upstream region. In *E*. *coli* K-12, a MarR-type transcriptional regulator, HpaR, which controls the transcription of the 4-hydroxyphenylacetate catabolic genes (*hpaGEDFHI*), binds to the intergenic region of *hpaR*−*hpaGEDFHI* and acts as a repressor of the *hpaG* operon and *hpaR*^40^. HpaR is thought to impede transcriptional elongation by binding to around +47 in the *hpaR* promoter region. The relatively similar position of the regulator binding sites suggests that the mode of transcriptional repression of *desB* by DesR may be similar to that of the autoregulation of *hpaR*. Although the HpaR dimer was suggested to form a repression loop by binding to two target sites^40^, a similar sequence of IR-B was not found upstream of *desB*. Therefore, a repression loop does not appear to be formed by DesR.

**Figure 7:**
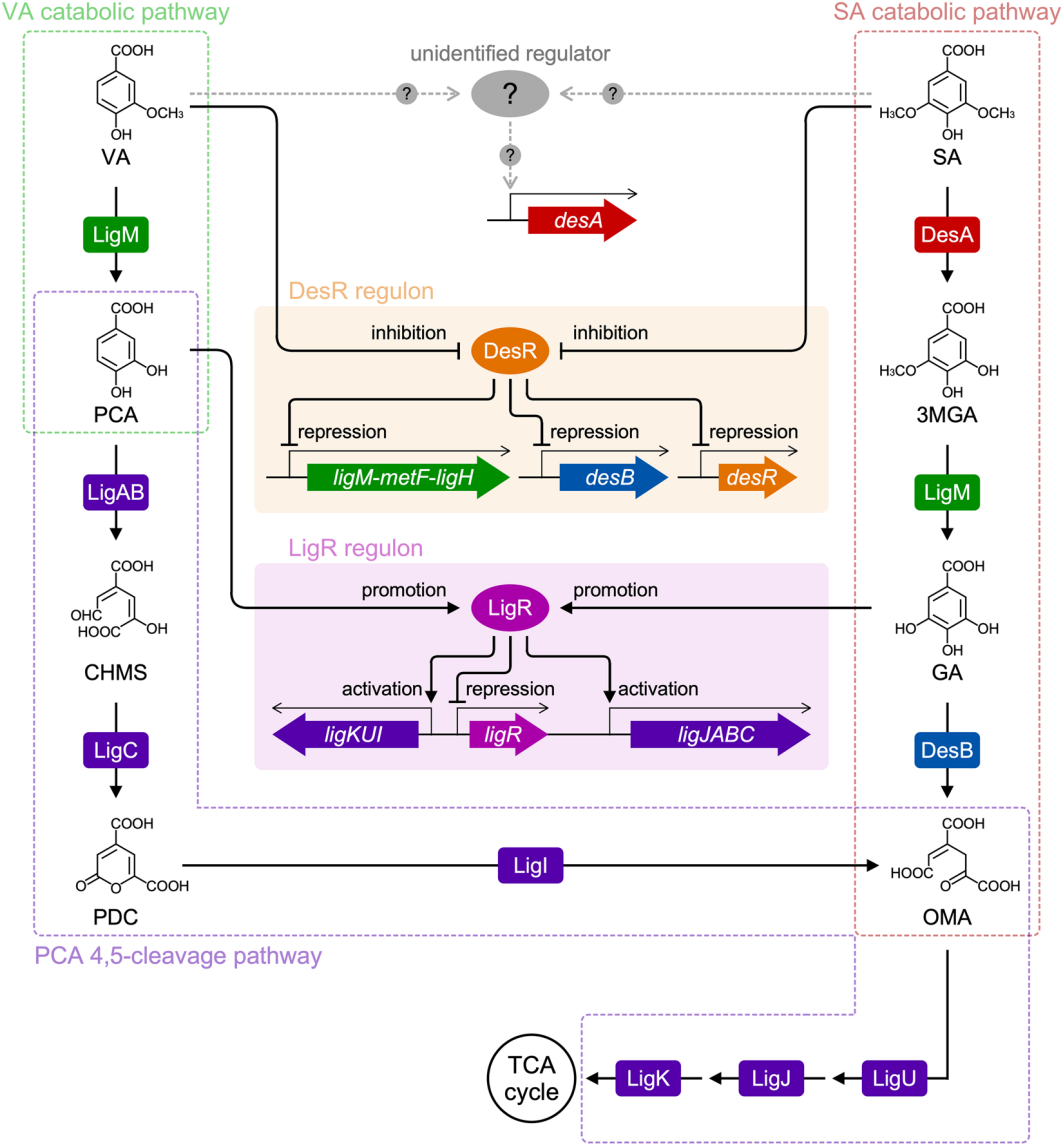
Proposed transcriptional regulatory system for VA and SA catabolism in *Sphingobium* sp. SYK-6. The regulation of *desB*, *ligM*, and *desR* by DesR and the regulation of the PCA 4,5-cleavage pathway genes by LigR are described in the Discussion section in detail. DesR regulon: *ligM*-*metF*-*ligH* operon, *desB*, and *desR*. LigR regulon: *ligJABC* operon, *ligKUI* operon, and *ligR*.

The relationship between VA/SA catabolism in SYK-6 and the transcriptional regulation of their catabolic genes can be described as follows (Fig. 7). In VA catabolism, the repression of *ligM* by DesR is released in the presence of VA and then the resulting LigM converts VA. The resultant PCA acts as an effector of a LysR-type transcriptional regulator, LigR, which activates the transcription of the PCA 4,5-cleavage pathway genes^33^. Via this pathway of enzymes, PCA is eventually catabolized into the tricarboxylic acid cycle. In SA catabolism, SA first induces the transcription of *desA* (which is controlled by an unidentified regulator that is different to DesR) and then the resulting DesA converts SA to 3MGA. Since the repression of *ligM* and *desB* by DesR is released in the presence of SA, the resulting LigM O-demethylates 3MGA to GA, and GA is then converted by DesB to an intermediate metabolite of the PCA 4,5-cleavage pathway. Because GA is another effector molecule of LigR^33^, GA-bound LigR is able to activate the transcription of the PCA 4,5-cleavage pathway genes. While SA catabolism branches into three different pathways from 3MGA (Fig. 1), the pathway involving *ligM* is essential because the generation of GA is indispensable for the activation of the PCA 4,5-cleavage pathway. Interestingly, the transcription of *desA* and *desB* was induced by VA despite these genes not being directly involved in VA catabolism (Fig. 2). This induction may contribute to the conversion of VA as DesA has some activity toward VA^28^. On the other hand, DesB is specific for the ring-cleavage of GA. Hence, this is a case where the inducer and the substrates being catabolized are not completely identical.

In the O demethylation of VA/3MGA and SA catalyzed by LigM and DesA, respectively, 5-methyl-H_4_folate is generated by transferring the methyl moiety of the methoxy-group of substrates to H_4_folate (Fig. 1). It is thought that in SYK-6 5-methyl-H_4_folate is metabolized via C_1_ metabolism involving 5,10-methylene-H_4_folate reductase (MetF), 5,10-methylene-H_4_folate dehydrogenase/5,10-methylene-H_4_folate cyclohydrolase (FolD), and 10-formyl-H_4_folate synthetase (LigH)^26^. C_1_-H_4_folate derivatives are essential for the synthesis of purines, *N*-formyl-methionyl tRNA, thymidylate, and methionine^41^. Since *ligM* constitutes an operon with *metF* and *ligH* (the *ligM*−*metF*−*ligH* operon)^12,16^, expression of this operon is essential for the growth of SYK-6. Based on the fact that *ligM* transcription is regulated by DesR, this regulator appears to have a crucial role in regulating not only the catabolism of lignin-derived aromatic compounds but also C_1_ metabolism. In SYK-6 cells, 5-methyl-H_4_folate acts as a methyl-group donor that is essential for methionine biosynthesis^28^. Therefore, SYK-6 exhibits auxotrophy for methionine when grown on a methoxy group-free substrate PCA as the sole carbon and energy source^26,28^, thus the supply of 5-methyl-H_4_folate in SYK-6 is completely dependent on the O demethylation step of VA and SA. Because other auxotrophies were not observed in SYK-6, 5,10-methylene-H_4_folate is also thought to be generated from the conversion of serine to glycine by serine hydroxymethyltransferase^12^.

The evolution of plants and differences in types of lignin in plant cell walls are known to be closely related. Gymnosperms containing G-lignin with minor amounts of H-lignin are thought to have appeared ca. 290–320 million years ago^42^. Angiosperms are thought to have emerged ca. 90–130 million years ago, and had gained the S-lignin biosynthetic pathway during their evolutionary process^42,43^. The *ligM* operon, regulated by DesR, appears to have arisen in bacteria to utilize aromatic compounds derived from G-lignin of gymnosperms (softwood lignin) as their carbon and energy source. Based on the appearance of S-lignin subsequent to G-lignin, bacteria may have acquired the SA catabolic system in addition to the VA catabolic system as an adaptation to changes in the types of lignin available in the environment. In SYK-6, following the establishment of the VA catabolic system linked to C_1_ metabolism regulated by DesR, *desA* may have been gained as a regulon that was controlled by a regulator other than DesR. In addition, *desB*, encoding a dioxygenase with an excellent catalytic ability for GA^31,44^, had appeared as part of the DesR regulon to efficiently utilize SA as a carbon source (Fig. 7).

In conclusion, the results obtained here are essential for an understanding of the whole regulatory system for lignin catabolism in bacteria, and will provide basic information not only for the engineering of bacteria capable of producing platform chemicals from lignin-derived compounds but also for the creation of bacterial sensors that specifically respond to VA and SA. However, identification of the transcriptional regulator of *desA* will be necessary to provide a more complete picture of the regulation of SA catabolism. There are no genes in the SYK-6 genome that show similarity with *desR* of SYK-6 or *vanR* of *A*. *baylyi*, *C*. *crescentus*, and *C*. *glutamicum*. This suggests that a regulator different from those reported is involved in the regulation of SA catabolism.

## Methods

### Bacterial strains, plasmids, primers, culture conditions, and chemicals

The strains and plasmids used in this study are listed in Table S3, and PCR primers are listed in Table S4. *Sphingobium* sp. SYK-6 and its mutants were grown at 30 °C with shaking (160 or 200 rpm) in LB, Wx medium containing 5 mM or 10 mM VA or SA, and Wx-SEMP containing 5 mM VA, PCA, SA, 3MGA, or GA. When necessary, the media for SYK-6 and its mutants and transformants were supplemented with 300 mg of carbenicillin/liter, 50 mg of kanamycin/liter, 12.5 mg of tetracycline/liter, or 12.5 mg of nalidixic acid/liter. *Escherichia coli* JM109 and *E*. *coli* NEB 10-beta were used for the cloning experiments. *E*. *coli* HB101 was used to transfer plasmids to SYK-6 by triparental mating. *E*. *coli* BL21(DE3) was used for the expression of *desR*. *E*. *coli* strains were grown at 30 °C or 37 °C with shaking (120 or 160 rpm) in LB. For cultures of cells carrying antibiotic resistance markers, the media for *E*. *coli* transformants were supplemented with 100 mg of ampicillin/liter or 25 mg of kanamycin/liter. VA, PCA, SA, and GA were purchased from the Tokyo Chemical Co., Ltd. (Tokyo, Japan) and 3MGA was purchased from Fluorochem Ltd. (Derbyshire, UK).

### Conversion of VA and SA by resting cells of SYK-6

Cells of SYK-6 grown in LB for 24 h were harvested by centrifugation (5,000 × *g*, 5 min, 4 °C) and then washed twice with Wx medium. The cells were resuspended in Wx medium, inoculated into 30 ml Wx-SEMP to an optical density at 600 nm (OD_600_) of 0.2, and incubated at 30 °C until the OD_600_ of the culture reached 0.5–0.6. Following the addition of 5 mM VA or SA, the cells were incubated for a further 6 h (cells in the exponential phase and approximately half of the substrate was consumed) as the inducing condition. For the non-inducing condition, the culture was incubated for a further 2 h (cells in the exponential phase) without adding any substrate. Cells were collected by centrifugation, washed twice with 50 mM Tris-HCl buffer (pH 7.5), and resuspended in the same buffer. The resultant cell suspensions were used as resting cells. Resting cells (OD_600_ = 5.0) were incubated with 200 µM VA or SA at 30 °C with shaking (1,500 rpm). Samples were collected at the start and after 5, 10, 20, and 30 min of incubation. The reactions were stopped by centrifugation (19,000 × *g*, 5 min, 4 °C). The supernatants were diluted 10-fold in distilled water, filtered using Millex-LG (Merck Millipore, Burlington, MA, USA), and analyzed using high-performance liquid chromatography (HPLC) (ACQUITY Ultra-Performance Liquid Chromatography System; Waters Corporation, Milford, MA, USA). HPLC analyses were performed using a TSKgel ODS-140HTP column (2.1 by 100 mm; Tosoh, Tokyo, Japan); the mobile phase was a mixture of water (90%) and acetonitrile (10%) containing 0.1% formic acid, at a flow rate of 0.5 ml/min. VA and SA were detected at 260.3 nm and 274.4 nm, with retention times of 1.6 and 1.8 min, respectively.

### Isolation of total RNA

Cells of SYK-6, SME021, and SME047 grown in LB for 24 h were harvested by centrifugation (5,000 × *g*, 5 min, 4 °C), washed twice with Wx medium, and resuspended in the same medium. The resulting cells were inoculated into 10 ml Wx-SEMP to an OD_600_ of 0.2, and incubated until the OD_600_ of the culture reached 0.5–0.6. Following the addition of 5 mM VA, PCA, SA, 3MGA, or GA, the cells were incubated for a further 6 h (VA, SA, and 3MGA) or 2 h (PCA and GA) as the inducing conditions (cells in the exponential phase and approximately half of the substrate was consumed). For the non-inducing condition, the culture was incubated for a further 2 h with no substrate added (cells in the exponential phase). Cells were collected by centrifugation and then total RNA was isolated from the cells using an Illustra RNAspin Mini RNA Isolation Kit (GE Healthcare, Buckinghamshire, UK). The RNA samples were treated with RNase-free DNase I (Takara Bio, Otsu, Japan), purified by phenol-chloroform extraction and ethanol precipitation, and finally dissolved in 10–20 µl diethylpyrocarbonate-treated water. The purity and concentration of resulting total RNAs were measured using a V630-BIO (Jasco, Tokyo, Japan) and the QuantiFluor RNA System (Promega, Madison, WI, USA), respectively.

### qRT-PCR

cDNAs were synthesized by reverse transcription using a PrimeScript II 1st Strand cDNA Synthesis Kit (Takara Bio). Total RNA (1 µg) was reverse transcribed using PrimeScript reverse transcriptase with random primers (6-mers). For each sample, a reverse transcriptase negative control was also prepared to verify no genomic DNA contamination had occurred. The synthesized cDNA was purified using a NucleoSpin Gel and PCR Clean-up Kit (Takara Bio) and eluted with 30 µl 5 mM Tris-HCl buffer (pH 8.5). qRT-PCR analyses were performed as previously described^33^, with specific primers listed in Table S4. For amplification of *ligM*, *desA*, and *desR*, specific primers were designed to amplify 5′-regions of these genes that remained in SME021, SME021, and SME047, respectively. The amounts (mol/µg total RNA) of each mRNA and 16S rRNA were measured using standard DNAs. To normalize the amount of mRNA in each sample, 16S rRNA was used as an internal standard (Table S5).

### LacZ reporter assay

To construct pRDBX, a 663-bp XhoI-PstI fragment containing the region 205-bp upstream of *desB* from pEVXH was inserted in front of the promoter-less *lacZ* of pPR9TZ. pRDBX was introduced into SYK-6 cells by electroporation. SYK-6 cells harboring pRDBX were grown in LB containing carbenicillin for 24 h, harvested by centrifugation, washed twice with Wx medium, and resuspended in the same medium. The resulting cells were inoculated into 10 ml Wx-SEMP containing carbenicillin to an OD_600_ of 0.2 and incubated until the OD_600_ of the culture reached 0.5–0.6. After the addition of 5 mM VA or SA, the cells were incubated for a further 6 h as the inducing conditions. For the non-inducing condition, the culture was incubated for a further 2 h without the addition of any substrate. Cells were harvested by centrifugation, washed twice with Wx medium, and resuspended in the same medium to an OD_600_ of 5.0. β-galactosidase activity was measured using V630-BIO and expressed as Miller units as previously described^45^.

### DNA affinity purification and protein identification

A 248-bp DNA fragment carrying the *desB* promoter region was amplified by PCR with biotin-labeled primers, as listed in Table S4. Biotin-labeled *desB* promoter fragments (desBp1 probe, 50 pmol) were bound to 0.6 mg streptavidin-conjugated magnetic beads (Dynabeads; Thermo Fisher Scientific, Waltham, MA, USA) according to the manufacturer’s instructions. SYK-6 cells grown in LB for 20 h were harvested by centrifugation, washed with TEGDI-0.1 buffer [10 mM Tris-HCl (pH 8.0), 1 mM EDTA, 10% glycerol, 1 mM DTT, 100 mM NaCl], resuspended in the same buffer, and then broken using an ultrasonic disintegrator (UD-201; Tomy Seiko, Tokyo, Japan). The resulting 3.5 ml of supernatant (ca. 1 mg of protein/ml) was mixed with 10 µl of 10 µg/µl salmon sperm DNA, 450 µl 80% glycerol, 450 µl 100 mM Tris-HCl buffer (pH 8.0) containing 500 mM KCl, 10 mM dithiothreitol, and 100 mg/l bovine serum albumin and then mixed with magnetic beads for 20 min. The magnetic beads were washed five times with 1 ml TEGDI-0.1 buffer then mixed with 50 µl TEN buffer [5 mM Tris-HCl (pH 8.0), 0.5 mM EDTA, 1 M NaCl]. The resulting supernatant was separated using SDS-PAGE, the gel was silver-stained, and detected spots were excised from the gel and destained. The samples were reduced by incubation for 1 h at 56 °C with 100 µl 100 mM NH_4_HCO_3_ containing 10 mM dithiothreitol, and then alkylated by shaking with 100 µl 100 mM NH_4_HCO_3_ containing 50 mM iodoacetamide for 40 min in the dark. Alkylated samples were shaken with 400 µl 50% methanol−10% acetic acid for 30 min four times, 400 µl 100 mM NH_4_HCO_3_ for 5 min, and then 400 µl acetonitrile for 5 min. After drying the samples in a vacuum centrifuge, in-gel tryptic digestion was performed using an XL-Tryp Kit (APRO SCIENCE, Tokushima, Japan). The peptide fragments obtained were subjected to desalting and concentration using Zip Tip (Merck Millipore). Mass spectra of the peptide samples were acquired by MALDI-TOF-MS analysis using Autoflex III (Bruker Daltonics, Billerica, MA, USA). Spectra were calibrated using standard peptides, bradykinin fragment 1–7 (756.3997 Da), ACTH fragment 18–39 (2,465.1989 Da), and insulin (5,730.6087 Da) (Sigma-Aldrich, St. Louis, MO, USA). To identify the protein, peptide masses were searched against the SYK-6 genome database using the Mascot search program (Matrix Science, London, UK).

### Construction of mutants

To construct pK19D12870 for the disruption of SLG_12870, an 879-bp fragment and a 784-bp fragment carrying the 5′ and 3′ regions of SLG_12870, respectively, were amplified by PCR from the total DNA of SYK-6 using specific primer sets, which are listed in Table S4. The resulting fragments were joined and amplified by overlapping PCR using 12870_T_F and 12870_B_R primers. After addition of adenine to the 3′ end of the fragment using Ex *Taq* DNA polymerase (Takara Bio) the resulting fragment was TA-cloned into a pT7Blue T-vector to obtain pT7D12870. The 1.7-kb HindIII-EcoRI fragment from pT7D12870 was inserted into the corresponding sites of pK19*mobsacB* to obtain pK19D12870. pK19D14170, for the disruption of SLG_14170, was constructed in a similar manner. Each of pK19D12870 and pK19D14170 was introduced into SYK-6 cells by triparental mating and the mutants were selected as previously described^46,47^. Disruption of each gene was examined by Southern hybridization analysis using the digoxigenin system (Roche, Mannheim, Germany). Detailed information on the mutants obtained is displayed in Fig. S2.

In order to construct a complementary plasmid, a 1.9-kb fragment carrying SLG_12870 was amplified from the total DNA of SYK-6 using 12870_T_F and 12870_B_R primers (Table S4). The resulting amplified fragment was inserted into the HindIII-EcoRI sites of pJB866 to obtain pJBdR (Table S3). pJB866 and pJBdR were independently introduced into SME047 and SYK-6 cells by electroporation, and the growth of transformants was measured as the following section using the medium containing tetracycline.

### Bacterial growth measurement

SYK-6, SME047, and SME058 cells were grown in 10 ml LB for 24 h, harvested by centrifugation (5,000 × *g*, 5 min, 4 °C), washed twice with Wx medium, and resuspended in 1 ml of the same medium. Cells were inoculated into 5 ml of the same medium containing 5 mM VA or SA to an OD_660_ of 0.2. Cell growth was periodically monitored by measuring the OD_660_ using a TVS062CA bio-photorecorder (Advantec, Tokyo, Japan) with shaking (60 rpm).

### Primer extension

Total RNA was isolated from SYK-6 cells grown in Wx medium containing 10 mM VA and SYK-6 cells harboring pKTDBP, which carried the *desB* promoter region, grown in Wx medium containing 10 mM SA. cDNAs were synthesized from total RNA (5 and 20 µg) with Beckman dye D4-labeled PEdesB (2 pmol) and D2-labeled PEligM (2 pmol), respectively, using a PrimeScript 1st Strand cDNA Synthesis Kit. The extended products were purified by phenol-chloroform extraction and ethanol precipitation then dissolved in CEQ Sample Loading Solution (Beckman Coulter, Fullerton, CA, USA) combined with DNA Size Standard Kit 400 (Beckman Coulter). Samples were analyzed utilizing a CEQ 2000XL genetic analysis system (Beckman Coulter).

### Expression of *desR* in *E. coli* and purification of DesR

A *desR*-coding region was amplified from the total DNA of SYK-6 using the desR_NdeI_F and desR_NdeI_R primers (Table S4). The 0.5-kb PCR product was cloned into pT7Blue to obtain pT1542, and then the 0.5 kb-NdeI-BamHI fragment from pT1542 was inserted into the corresponding sites of pCold I generating pCIdR. *E*. *coli* BL21(DE3) cells harboring pCIdR were grown in LB containing ampicillin for 12 h at 37 °C, and the culture was inoculated into the same fresh medium (final concentration, 1%). Once the OD_600_ of the culture had reached 0.5–0.6, the culture was left for 30 min at 16 °C. Gene expression was then induced by incubation for 24 h at 16 °C and the addition of 1 mM isopropyl-β-D-thiogalactopyranoside. The cells were harvested by centrifugation (5,000 × *g*, 5 min, 4 °C), washed twice with 50 mM Tris-HCl buffer (pH 7.5) containing 500 mM NaCl and 40 mM imidazole (buffer A), resuspended in buffer A, and broken using an ultrasonic disintegrator. The supernatant obtained by centrifugation (19,000 × *g*, 15 min, 4 °C) was applied to a His Spin Trap TALON (GE Healthcare) previously equilibrated with buffer A. After centrifugation (100 × *g*, 1 min, 4 °C), samples were washed five times with buffer A and then His tag-fused DesR was eluted with 50 mM Tris-HCl buffer (pH 7.5) containing 500 mM NaCl and 150 mM imidazole. Purified DesR was subjected to desalting and concentration by centrifugal filtration using an Amicon Ultra 10k filter unit (Merck Millipore). Before the assay, insoluble aggregates in the DesR solution were removed by centrifugal filtration using an Ultrafree-MC filter (Merck Millipore). Protein concentration was determined by the Bradford method^48^ with bovine serum albumin as the standard (Bio-Rad Laboratories). The purified protein sample was analyzed by SDS-12% PAGE.

### Size exclusion chromatography of DesR

Purified DesR (ca. 400 µg) was subjected to size exclusion chromatography using a Superdex 200 Increase 10/300GL column (GE Healthcare) with the BioAssist eZ system (Tosoh). Elution was performed with 10 mM KH_2_PO_4_-K_2_HPO_4_ buffer (pH 7.4) containing 140 mM NaCl at a flow rate of 1.0 ml/min. Gel-filtration calibration kits (HMW and LMW; GE Healthcare) were used as the molecular mass standard.

### EMSA

EMSAs for cell extracts and purified DesR were performed using a DIG Gel Shift Kit (2nd generation) (Roche). For preparation of cell extracts, SYK-6, SME047, and SME058 cells were grown in LB for 24 h, harvested by centrifugation (5,000 × *g*, 5 min, 4 °C), washed twice with Wx medium, and resuspended in the same medium. The resulting cells were inoculated into 10 ml Wx-SEMP to an OD_600_ of 0.2, and incubated until the OD_600_ of the culture reached 0.5–0.6. Following the addition of 5 mM VA or SA, the cells were incubated for a further 6 h as the inducing conditions. For the non-inducing condition, the culture was incubated for a further 2 h with no substrate added. The cells were harvested by centrifugation (5,000 × *g*, 5 min, 4 °C), washed twice with Wx medium, resuspended in the same medium, and broken using an ultrasonic disintegrator. The supernatants were obtained by centrifugation (19,000 × *g*, 15 min, 4 °C) as the cell extracts.

DNA fragments used for probes were amplified from the total DNA of SYK-6 by PCR, using the specific primers listed in Table S4. Purified fragments were labeled at their 3′ end with digoxigenin-11-ddUTP using terminal transferase. The DNA–protein binding reactions were carried out at 20 °C for 20 min in a final volume of 10 µl mixture containing cell extracts (4 µg of protein) or purified DesR [10 ng (0.24 pmol dimer) or 100 ng (2.4 pmol dimer)], 5 fmol digoxigenin-labeled probe, 1 µg poly-[d(I-C)], and binding buffer [20 mM HEPES, 1 mM EDTA, 10 mM (NH_4_)_2_SO_4_, 1 mM dithiothreitol, 0.2% (w/v) Tween 20, 30 mM KCl, pH 7.6]. To examine the association of DesR with effector molecules, 1 µl VA or SA (0.05, 0.5, 5, or 50 mM) was added to the reaction mixture. Reaction mixtures were separated on a 5% native polyacrylamide gel and signals were detected using a CSPD-based chemiluminescence detection system (Roche)^33^.

## Supplementary information

Supplementary information

## Data Availability

All data supporting this study are available within the article and its Supplementary Information files or are available from the corresponding author upon request.
