# Supplementary material for: Regulation of vanillate and syringate catabolism by a MarR-type transcriptional regulator DesR in Sphingobium sp. SYK-6
Source: Sci Rep. 2019 Dec 2;9:18036. doi: 10.1038/s41598-019-54490-7 (PMC6888825; doi:10.1038/s41598-019-54490-7)
Supplement: Supplementary file 1 — Supplementary information [file 41598_2019_54490_MOESM1_ESM.pdf]

Supplementary information

Regulation of vanillate and syringate catabolism by a MarR-type transcriptional regulator DesR in *Sphingobium* sp. SYK-6

Takuma Araki,[1,2] Shusuke Umeda,[1] Naofumi Kamimura,[1] Daisuke Kasai,[1] Shuta Kumano,[1] Tomokuni Abe,[1] Chika Kawazu,[1] Yuichiro Otsuka,[2] Masaya Nakamura,[2] Yoshihiro Katayama,[3] Masao Fukuda,[1,†] and Eiji Masai[1*]

[1)] *Department of Bioengineering, Nagaoka University of Technology, Nagaoka, Niigata 940-2188, Japan*

[2)] *Forestry and Forest Products Research Institute, Tsukuba, Ibaraki 305-8687, Japan*

[3)] *College of Bioresource Sciences, Nihon University, Fujisawa, Kanagawa 252-0880, Japan*

Key words: lignin; vanillate; syringate; transcriptional regulation; *Sphingobium*

[*]Corresponding author:

Eiji Masai

Department of Bioengineering, Nagaoka University of Technology, Nagaoka, Niigata 940-2188, Japan

E-mail emasai@vos.nagaokaut.ac.jp

Tel. +81 258479428

[†]Present address:

Department of Biological Chemistry, Chubu University, Kasugai, Aichi 487-8501, Japan

Contents list

Supplementary tables: Table S1-S5

Supplementary figures: Fig. S1-S7

References for Supplementary information

Table S1. BLAST search of SLG_12870 against the NCBI non-redundant database

RefSeq ID or Accession No.	E-value scores	Description
OJY67212.1	1.01E-79	MarR family transcriptional regulator [*Sphingobium* sp. 66-54]
WP_100283329.1	7.08E-71	MarR family transcriptional regulator [*Sphingomonas* sp. Cra20]
WP_066804699.1	4.04E-70	MarR family transcriptional regulator [*Sphingomonas asaccharolytica*]
WP_066818941.1	1.41E-69	MarR family transcriptional regulator [*Sphingomonas mali*]
WP_091149011.1	1.02E-64	MarR family transcriptional regulator [*Novosphingobium* sp. CF614]
OYY70249.1	3.44E-64	MarR family transcriptional regulator [*Sphingomonas* sp. 28-63-12]
WP_067615223.1	6.27E-64	MarR family transcriptional regulator [*Novosphingobium* sp. FSW06-99]
WP_067743213.1	8.30E-63	MarR family transcriptional regulator [*Novosphingobium* sp. Fuku2-ISO-50]
PIX64619.1	1.33E-62	MarR family transcriptional regulator [*Sphingomonadales bacterium* CG_4_10_14_3_um_filter_58_15]
WP_120252661.1	4.29E-62	MarR family transcriptional regulator [*Sphingobium* sp. YG1]
AJP73374.1	2.07E-61	MarR family transcriptional regulator [*Sphingomonas hengshuiensis*]
WP_067198499.1	2.16E-61	MarR family transcriptional regulator [*Sphingorhabdus* sp. M41]
WP_066863135.1	2.47E-61	MarR family transcriptional regulator [*Sphingobium* sp. TCM1]
WP_028639593.1	3.86E-61	MarR family transcriptional regulator [*Novosphingobium acidiphilum*]
RYD67871.1	5.13E-61	MarR family transcriptional regulator [*Sphingomonadales bacterium*]
WP_056685192.1	6.19E-61	MarR family transcriptional regulator [*Sphingobium* sp. Leaf26]
OJU17880.1	1.00E-60	MarR family transcriptional regulator [*Sphingomonas* sp. 66-10]
WP_127706381.1	3.64E-60	MarR family transcriptional regulator [*Novosphingobium* sp. FSY-9]

E-value scores of the top hit genes in database are listed.

Table S2. BLAST search of SLG_14170 against the NCBI non-redundant database

RefSeq ID or Accession No.	E-value scores	Description
BBE33967.1	1.01E-169	LysR family transcriptional regulator [*Sphingosinicella microcystinivorans*]
WP_116841920.1	5.44E-145	LysR family transcriptional regulator [*Sphingomonas* sp. CF311]
WP_034156651.1	5.94E-145	LysR family transcriptional regulator [*Sphingomonas* sp. ERG5]
WP_086127351.1	1.93E-143	LysR family transcriptional regulator [*Sphingomonas* sp. IBVSS2]
WP_010402354.1	5.28E-143	LysR family transcriptional regulator [*Sphingomonas echinoides*]
WP_066858994.1	1.02E-141	LysR family transcriptional regulator [*Sphingobium* sp. TCM1]
WP_098104023.1	1.72E-141	LysR family transcriptional regulator [*Novosphingobium* sp. PC22D]
WP_031310277.1	5.72E-141	LysR family transcriptional regulator [*Sphingobium* sp. C100]
EQB12782.1	1.41E-140	LysR family transcriptional regulator [*Sphingobium lactosutens* DS20]
WP_119761974.1	1.67E-140	LysR family transcriptional regulator [*Sphingomonas* sp. K2R01-6]
PDH63925.1	1.71E-140	LysR family transcriptional regulator [*Sphingomonadaceae bacterium* MED-G03]
WP_009823557.1	3.72E-140	LysR family transcriptional regulator [*Sphingomonas* sp. SKA58]
ETI59073.1	6.40E-140	LysR family transcriptional regulator [*Sphingobium* sp. C100]
WP_062732812.1	1.24E-139	LysR family transcriptional regulator [*Sphingobium abikonense*]
WP_114952386.1	2.58E-139	LysR family transcriptional regulator [*Sphingomonadaceae bacterium* KCTC 52780]

E-value scores of the top hit genes in database are listed.

Table S3. Strains and plasmids used in this study

Strain or plasmid	Relevant characteristic(s)[a]	Reference or source
Sphingobium sp.		
SYK-6	Wild type; Nal^r Sm^r	1
SME021	SYK-6 derivative; *ligM*::*bla desA*::*kan*; Nal^r Sm^r Cb^r Km^r	2
SME047	SYK-6 derivative; ΔSLG_12870 (*desR*); Nal^r Sm^r	This study
SME058	SYK-6 derivative; ΔSLG_14170; Nal^r Sm^r	This study
E. coli		
JM109	*recA1 supE44 endA1 hadR17*(r_K^- m_K^+) *gyrA96 relA1 thi-1* Δ(*lac-proAB*) F′[*traD36 proAB*$^+$ *lacI*^{q} *lacZ*ΔM15]	3
NEB 10-beta	Δ(*ara-leu*) *7697 araD139 fhuA* Δ*lacX74 galK16 galE15 e14*-ϕ*80*d*lacZ*Δ*M15 recA1 relA1 endA1 nupG rpsL* (Sm^r) *rph spoT1* Δ(*mrr-hsdRMS-mcrBC*)	New England Biolabs
HB101	*recA13 supE44 hsd20 ara-14 proA2 lacY1 galK2 rpsL20 xyl-5 mtl-1*	4
BL21(DE3)	F$^-$ *ompT hsdS*$_B$(r_B^- m_B^-) *gal dcm* (DE3); T7 RNA polymerase gene under control of the *lacUV5* promoter	5
Plasmids		
pKT230	Broad-host-range vector; Km^r	6
pRK2013	Tra$^+$ Mob$^+$ ColE1 replicon; Km^r	7
pT7Blue	Cloning vector; T7 promoter; Ap^r	Novagen
pBluescript II KS(+)	Cloning vector; Ap^r	8
pColdI	Expression vector; *cspA* promoter; Ap^r	Takara Bio
pK19*mobsacB*	*oriT sacB*; Km^r	9
pJB866	RK2 broad-host-range expression vector; Tc^r P_m *xylS*	10
pPR9TT	Translational fusion LacZ reporter vector; Ap^r Cm^r	11
pQF50	Broad-host-range transcriptional fusion vector containing a promoterless *lacZ*; Ap^r	12
pPR9TZ	pPR9TT with a 3.6-kb SmaI-ScaI fragment containing *lacZ* from pQF50 replacing the 3.2-kb BamHI fragment	13
pEVXH	KS(+) with a 3.1-kb XhoI-EcoRV fragment carrying *desB*	14
pRDBX	pPR9TZ with a 0.7-kb XhoI-PstI fragment carrying the *desB* promoter region from pEVXH	This study
pJBdR	pJB866 with a 1.9-kb PCR amplified HindIII-EcoRI fragment carrying *desR*	This study
pBDBP	KS(+) with a 1.4-kb PstI fragment of pEVXH	This study
pKTDBP	pKT230 with a 1.2-kb EcoRI fragment carrying the *desB* promoter region of pBDBP	This study
pT7D12870	pT7Blue with a 1.7-kb PCR amplified fragment carrying *desR*	This study
pK19D12870	pK19*mobsacB* with a 1.7-kb HindIII-EcoRI fragment carrying *desR* from pT7DdesR	This study
pK19D14170	pK19*mobsacB* with a 1.7-kb PCR amplified fragment carrying SLG_14170	This study
pT1542	pT7Blue with a 0.5-kb PCR amplified fragment carrying *desR*	This study
pCIdR	pColdI with a 0.5-kb NdeI-BamHI fragment carrying *desR* from pT1542	This study

[a]Nal^r, Sm^r, Cb^r, Km^r, Ap^r, Tc^r, and Cm^r, resistance to nalidixic acid, streptomycin, carbenicillin, kanamycin, ampicillin, tetracycline, and chloramphenicol, respectively.

Table S4. Primers used in this study

Purposes	Primers	Sequences (5′ to 3′)
qRT-PCR analysis	16S_F	GCGCAGAACCTTACCAACGT
	16S_R	AGCCATGCAGCACCTGTCA
	desA_F	GCCTTCGCCTTCCTCAACTA
	desA_R	CACCGGAACCCACTGCTT
	ligM_F	ACGTACTGCTTCGCCTTGTTG
	ligM_R	GCTCTCCGACACGATGATCA
	desB_F	TTTCGAGCATTATTCGCATTTC
	desB_R	TCCGCAGGCGAATATTCCT
	desR_F	TTGGAAGCAAGAGATAGACAGCAT
	desR_R	TCGTCCGCGCATTCAAG
Isolation of proteins bound to the *desB* promoter	bt-desB_F[a]	bt-GCCGTTCCCCTCTCAGGC
	bt-desB_R[a]	bt-AAACCGCCGATGATCTTTGC
Gene disruption and complementation	12870_T_F	CGAAGCTTAGATTTCATAGAGGCACAC (HindIII)
	12870_T_R	CTTGGTGAAGCGGGATTTTGAAGGTTTCCGTGAAGTGA
	12870_B_F	AAATCCCGCTTCACCAAG
	12870_B_R	CGGAATTCAGCGAGCGATTGATCTTCTC (EcoRI)
	14170_T_F	AGTTCTAGATCGCGCTGTTCCT (XbaI)
	14170_T_R	CCGAGGCAATGTAGAAAGTGCTGGAAAACCTCGATATGGCG
	14170_B_F	GCACTTTCTACATTGCCTCGG
	14170_B_R	ATGAGAATTCCCGTCTGCCC (EcoRI)
Primer extension	PEdesB[b]	D4-GTCATTGTAGACGTAGAAGGTCACG
	PEligM[c]	D2-GAAGAGCACTGCCGAATTGC
Protein expression	desR_NdeI_F	AAGACATATGGAAGCAAGAGATAG (NdeI)
	desR_NdeI_R	AATCTATTCGTGGATACGGC
EMSAs	desBp1_F	GCCGTTCCCCTCTCAGGC
	desBp2_F	ACAGAGCTTGGCCGTG
	desBp3_F	TTCACGCACTGCCCATTCG
	desBp4_F	AGAAGTTCGGCAGTCTGC
	desBp_R	AAACCGCCGATGATCTTTGC
	ligMp1_F	GGGGCACGAAATTTTCGTTTG
	ligMp1_R	GTCGACCATGTGGTGGGT
	ligMp2_F	ATGGACGAGGGCTTCACG
	ligMp2_R	ACGCCCGGAAACATACGAAT
	desAp1_F	CTGCAGGATGTGCGCC
	desAp1_R	GTGAATGCCAGCCCCAAA
	desAp2_F	GCATGACCTTTCAATTGTGCG
	desAp2_R	AAGTGAATGCCGGTGCTGAT
	desAp3_F	AGATCATTCGCCGCGCA
	desAp3_R	ATGGCTTCATGCTGCACC
	desAp4_F	ACCGGCGCCGATGATGC
	desAp4_R	TGGTTGAAGAGCACGGCGG
	desRp1_F	ACCAGGTTCGGAATGTCGCA
	desRp1_R	GGAAGCCGATGATGTCGTCG
	desBp5_IR-B_R	GAACTTCTGTATGTGACACAAACGAAATTTT
	desBp5_IR-B_m2nt_R	GAACTTCTGTATGTGACACAAGTGAAATTTT
	desBp5_IR-B_m3nt_R	GAACTTCTGTATGTGACGTGAACGAAATTTT
	desBp5_IR-B_m6nt_R	GAACTTCTGTATGTGATGTGAGTGAAATTTT
	desBp6_IR-B_F	AAAATTTCGTTTGTGTCACATACAGAAGTTC
	desBp6_IR-B_m2nt_F	AAAATTTCACTTGTGTCACATACAGAAGTTC
	desBp6_IR-B_m3nt_F	AAAATTTCGTTCACGTCACATACAGAAGTTC
	desBp6_IR-B_m6nt_F	AAAATTTCACTCACATCACATACAGAAGTTC
	ligMp3_IR-M_R	GAATTTTTGTATGTTACACAAACGAAAATTT
	ligMp3_IR-M_m2nt_R	GAATTTTTGTATGTTACACAAGTGAAAATTT
	ligMp3_IR-M_m3nt_R	GAATTTTTGTATGTTACGTGAACGAAAATTT
	ligMp3_IR-M_m5nt_R	GAATTTTTGTATGTTACGTGAGTGAAAATTT
	ligMp4_IR-M_F	AAATTTTCGTTTGTGTAACATACAAAAATTC
	ligMp4_IR-M_m2nt_F	AAATTTTCACTTGTGTAACATACAAAAATTC
	ligMp4_IR-M_m3nt_F	AAATTTTCGTTCACGTAACATACAAAAATTC

	ligMp4_IR-M_m5nt_F	AAATTTTCACTCACGTAACATACAAAAATTC
	desRp2_IR-R_F	AAGTAATTGTATGCTACGCTTACGAAATCGA
	desRp2_IR-R_m5nt_F	AAGTAATTACGTATTACGCTTACGAAATCGA

[a] Primers labeled with biotin at the 5′ end.
[b] A primer labeled with Beckman Dye D4 at the 5′ end.
[c] A primer labeled with Beckman Dye D2 at the 5′ end.

Table S5. The amount of 16S rRNA measured by qRT-PCR analyses

Strain	Induction	pmol/μg total RNA
Wild type	none	0.547 ± 0.046
	VA	0.550 ± 0.11
	PCA	0.709 ± 0.10
	SA	0.520 ± 0.068
	3MGA	0.546 ± 0.037
	GA	0.615 ± 0.053
SME047	none	0.803 ± 0.044
	VA	0.671 ± 0.084
	SA	0.518 ± 0.031
SME021	none	0.763 ± 0.12
	VA	0.650 ± 0.049
	SA	0.722 ± 0.14

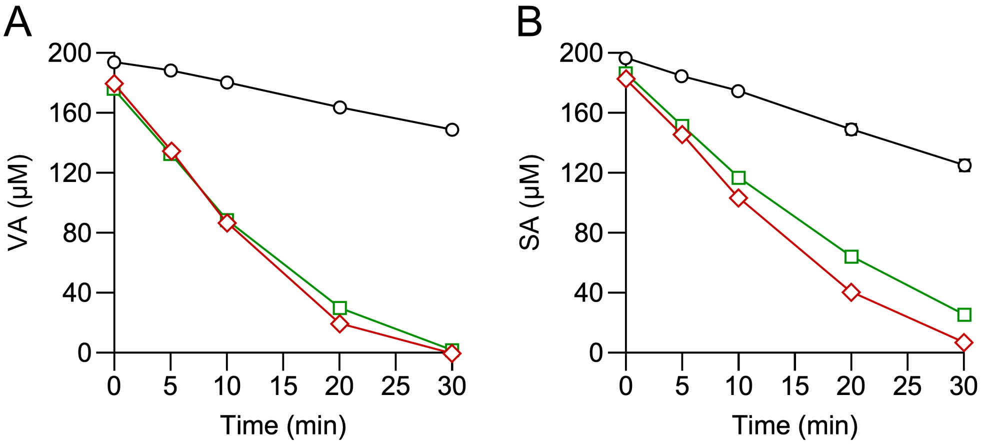

Fig. S1. Conversion of VA and SA by resting cells of SYK-6. Resting cells of SYK-6 grown in Wx-SEMP (black circles), Wx-SEMP plus VA (green squares), and Wx-SEMP plus SA (red diamonds) were incubated with 200 µM VA (A) and SA (B). The amount of VA and SA in the cultures was periodically monitored using HPLC. Each value is the average ± the standard deviation (error bars) of three independent experiments. Error bars are hidden behind the symbols.

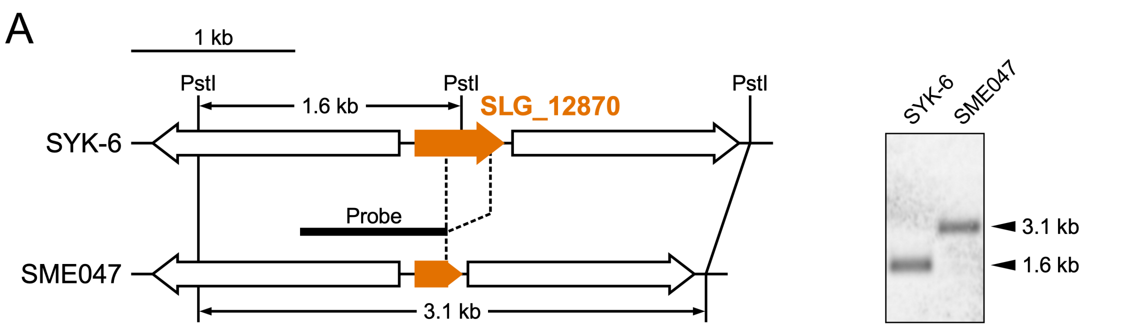

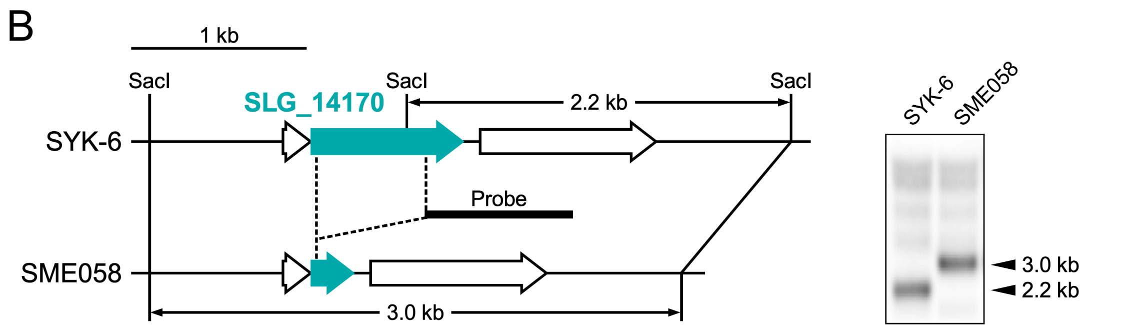

Fig. S2. Disruption of SLG_12870 and SLG_14170 in SYK-6. (A and B) The panels on the left show schematic representations of the disruption of SLG_12870 and SLG_14170, respectively. The disruption of SLG_12870 and SLG_14170 was examined by Southern hybridization of the PstI-digests of the total DNA of SME047 and the SacI-digests of the total DNA of SME058 using the probes shown in the panels on the left. In SME047 and SME058, the regions at positions 188–453 of SLG_12870 (519 bp from TTG to TAG) and 33–667 of SLG_14170 (888 bp from GTG to TAG) were deleted, respectively.

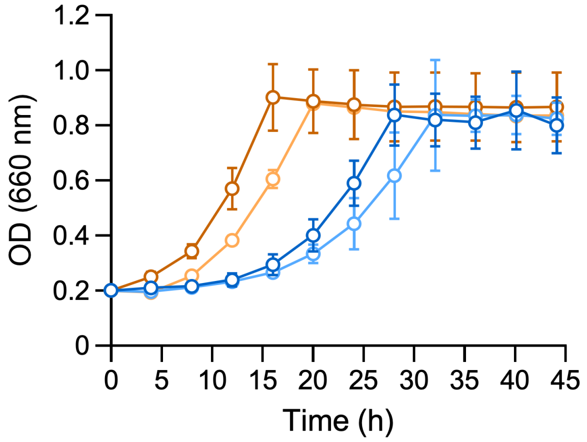

Fig. S3. Effects of the introduction of SLG_12870 in SME047 and SYK-6 on their growth on VA. Cells of SYK-6 harboring pJB866 (vector, dark blue), SYK-6 harboring pJBdR (light blue), SME047 harboring pJB866 (dark orange), and SME047 harboring pJBdR (light orange) were incubated in Wx medium containing 5 mM VA. OD_{660} was periodically monitored. The growth of SME047(pJB866) and SYK-6(pJB866) on VA was slower than that of SME047 and SYK-6 on VA (Fig. 3) probably due to the presence of tetracycline in the media for the transformants. Each value is the average ± the standard deviation (error bars) of three independent experiments.

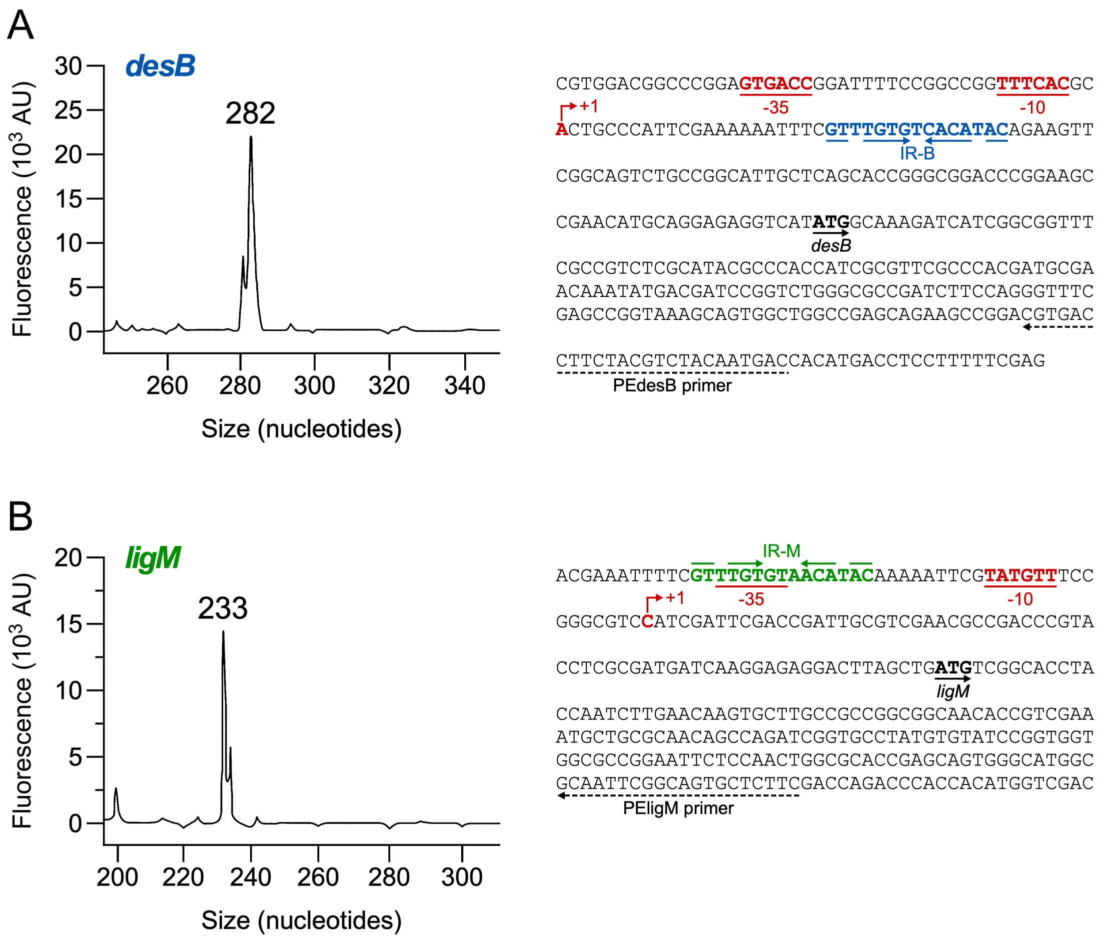

Fig. S4. Determination of the transcription start sites of *desB* and *ligM* by primer extension. (A) The transcription start site of *desB* was determined using total RNA isolated from SYK-6 cells harboring pKTDBP grown in Wx-10 mM SA and the PEdesB primer. (B) The transcription start site of *ligM* was determined using total RNA isolated from SYK-6 cells grown in Wx-10 mM VA and the PEligM primer. The panels on the left show the analysis of extended products using a CEQ 2000XL Genetic Analysis System. The panels on the right show the nucleotide sequences of the *desB* and *ligM* promoter regions. The initiation codons of *desB* and *ligM* are shown by arrows. The transcription start sites are shown by bent arrows (+1). Putative −35 and −10 regions are underlined. The inverted repeat sequences (IR-B and IR-M) are indicated by convergent arrows. Dotted arrows indicate the position of the PEdesB and PEligM primers.

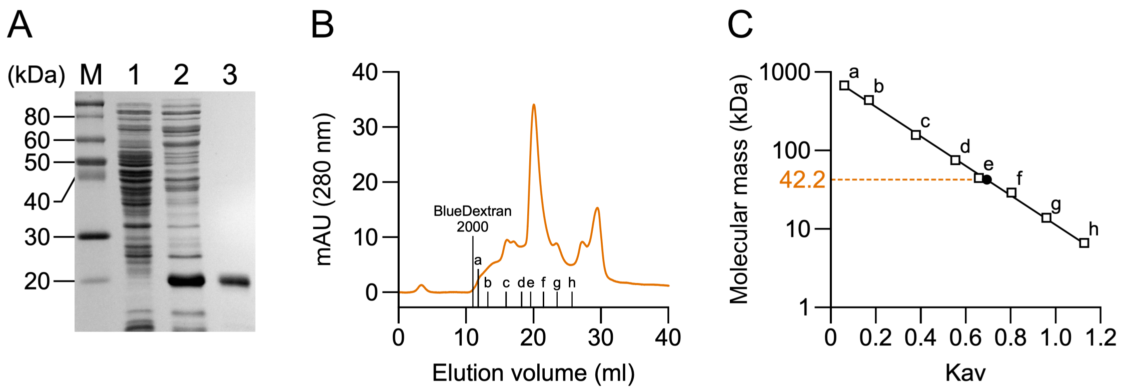

Fig. S5. Purification and molecular mass determination of DesR. (A) Expression of *desR* in *E. coli* BL21(DE3) and the purification of DesR. Proteins were separated using SDS-12% PAGE and stained with Coomassie Brilliant Blue. Lanes: M, molecular mass markers; 1, cell extract of *E. coli* BL21(DE3) harboring pCold I (vector) (10 μg protein); 2, cell extract of *E. coli* BL21(DE3) harboring pCIdR (10 μg protein); 3, purified DesR (1 μg protein). (B) Size exclusion chromatography of DesR. Standard proteins: (a) thyroglobulin (669 kDa), (b) ferritin (440 kDa), (c) aldolase (158 kDa), (d) conalbumin (75.0 kDa), (e) ovalbumin (44.0 kDa), (f) carbonic anhydrase (29.0 kDa), (g) ribonuclease A (13.7 kDa), and (h) aprotinin (6.5 kDa). The void volume of the column was determined from the retention time of Blue Dextran. (C) Estimation of the molecular mass of DesR (black circle). The calibration line was determined using standard proteins.

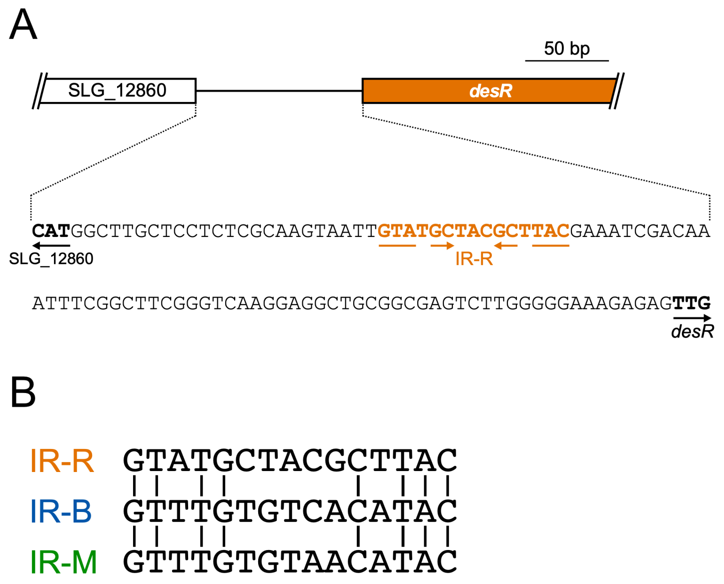

Fig. S6. IR-R present in the upstream region of *desR*. (A) Nucleotide sequence of the SLG_12860–*desR* intergenic region. The initiation codons of SLG_12860 and *desR* are shown by arrows. The inverted repeat sequences (IR-R) are shown by convergent arrows. (B) Nucleotide sequence alignment between the sequences of IR-R, IR-B, and IR-M. Vertical lines indicate conserved residues.

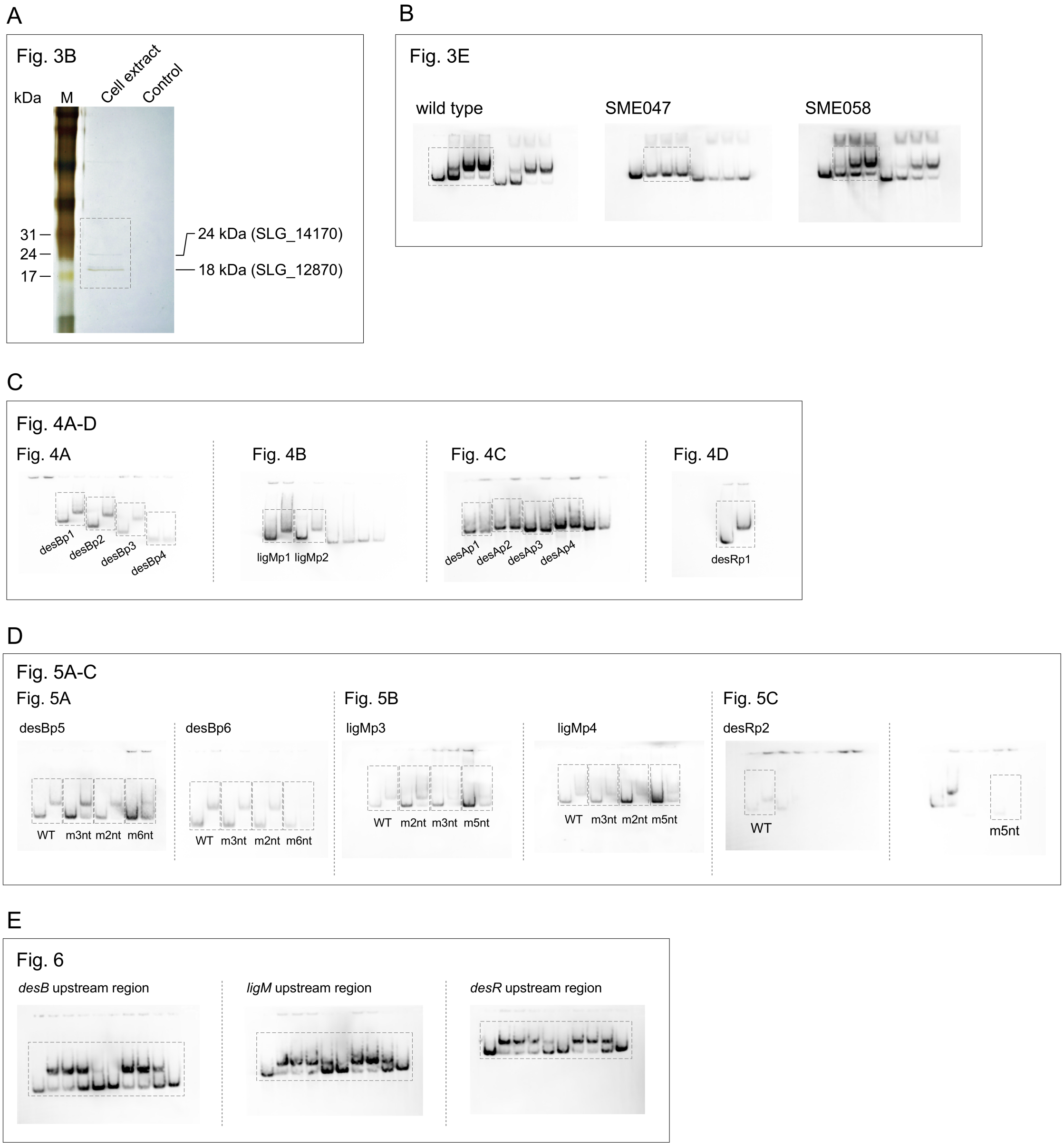

Fig. S7. Uncropped images shown in Fig. 3B (A), Fig. 3E (B), Fig. 4 (C), Fig. 5 (D), and Fig. 6 (E).

References

1 Katayama, Y. *et al.* Cloning and expression of *Pseudomonas paucimobilis* SYK-6 genes involved in the degradation of vanillate and protocatechuate in *P. putida*. *Mokuzai Gakkaishi* **33**, 77-79 (1987).

2 Abe, T., Masai, E., Miyauchi, K., Katayama, Y. & Fukuda, M. A tetrahydrofolate-dependent *O*-demethylase, LigM, is crucial for catabolism of vanillate and syringate in *Sphingomonas paucimobilis* SYK-6. *J Bacteriol* **187**, 2030-2037, doi:10.1128/jb.187.6.2030-2037.2005 (2005).

3 Yanisch-Perron, C., Vieira, J. & Messing, J. Improved M13 phage cloning vectors and host strains: nucleotide sequences of the M13mp18 and pUC19 vectors. *Gene* **33**, 103-119 (1985).

4 Bolivar, F. & Backman, K. Plasmids of *Escherichia coli* as cloning vectors. *Methods Enzymol* **68**, 245-267 (1979).

5 Studier, F. W. & Moffatt, B. A. Use of bacteriophage T7 RNA polymerase to direct selective high-level expression of cloned genes. *J Mol Biol* **189**, 113-130 (1986).

6 Bagdasarian, M. *et al.* Specific-purpose plasmid cloning vectors. II. Broad host range, high copy number, RSF1010-derived vectors, and a host-vector system for gene cloning in *Pseudomonas*. *Gene* **16**, 237-247 (1981).

7 Figurski, D. H. & Helinski, D. R. Replication of an origin-containing derivative of plasmid RK2 dependent on a plasmid function provided in trans. *Proc Natl Acad Sci U S A* **76**, 1648-1652, doi:10.1073/pnas.76.4.1648 (1979).

8 Short, J. M., Fernandez, J. M., Sorge, J. A. & Huse, W. D. λ ZAP: a bacteriophage λ expression vector with *in vivo* excision properties. *Nucleic Acids Res* **16**, 7583-7600, doi:10.1093/nar/16.15.7583 (1988).

9 Schäfer, A. *et al.* Small mobilizable multi-purpose cloning vectors derived from the *Escherichia coli* plasmids pK18 and pK19: selection of defined deletions in the chromosome of *Corynebacterium glutamicum*. *Gene* **145**, 69-73 (1994).

10 Blatny, J. M., Brautaset, T., Winther-Larsen, H. C., Karunakaran, P. & Valla, S. Improved broad-host-range RK2 vectors useful for high and low regulated gene expression levels in gram-negative bacteria. *Plasmid* **38**, 35-51, doi:10.1006/plas.1997.1294 (1997).

11 Santos, P. M., Di Bartolo, I., Blatny, J. M., Zennaro, E. & Valla, S. New broad-host-range promoter probe vectors based on the plasmid RK2 replicon. *FEMS Microbiol Lett* **195**, 91-96, doi:10.1111/j.1574-6968.2001.tb10503.x (2001).

12 Farinha, M. A. & Kropinski, A. M. Construction of broad-host-range plasmid vectors for easy visible selection and analysis of promoters. *J Bacteriol* **172**, 3496-3499, doi:10.1128/jb.172.6.3496-3499.1990 (1990).

13 Kamimura, N. *et al.* Regulatory system of the protocatechuate 4,5-cleavage pathway genes essential for lignin downstream catabolism. *J Bacteriol* **192**, 3394-3405, doi:10.1128/JB.00215-10 (2010).

14 Kasai, D., Masai, E., Miyauchi, K., Katayama, Y. & Fukuda, M. Characterization of the gallate dioxygenase gene: three distinct ring cleavage dioxygenases are involved in syringate degradation by *Sphingomonas paucimobilis* SYK-6. *J Bacteriol* **187**, 5067-5074, doi:10.1128/jb.187.15.5067-5074.2005 (2005).
